# Extraction of Time-Varying Signals in GNSS and Geophysical Interpretation: Methods, Advances, and Challenges

**DOI:** 10.3390/s26175557

**Published:** 2026-09-01

**Authors:** Xiangjun Li, Shuguang Wu, Houpu Li, Bing Liu, Shaofeng Bian, Yuefan He

**Affiliations:** 1 School of Electrical Engineering, Naval University of Engineering, Wuhan 430033, China; 1920191191@nue.edu.cn (X.L.); d24381107@nue.edu.cn (B.L.); 0910051014@nue.edu.cn (S.B.); 2 Department of Basic Courses, Naval University of Engineering, Wuhan 430033, China; 3 Faculty of Geomatics, Lanzhou Jiaotong University, Lanzhou 730070, China; heyuefan@whu.edu.cn

**Keywords:** GNSS coordinate time series, time-varying signal extraction, environmental loading deformation, geophysical interpretation, multi-source data fusion

## Abstract

**Highlights:**

**What are the main findings?**

- Maximum Likelihood Estimation (MLE) has become the standard for GNSS linear trend extraction because it jointly estimates trend parameters and stochastic noise covariance; by doing so, it corrects the up to 5–10-fold underestimation of velocity uncertainty that occurs when temporally correlated (colored) noise is present but a white-noise covariance is assumed.
- In large-sample benchmark tests on the CMONOC network reported by Wu et al., Variational Mode Decomposition (VMD) yields positive residual RMS improvement at 97.9% of stations (mean 69.8%), outperforming SSA (90.5% of stations); these results originate from the authors’ prior work and await independent replication on other networks before generalization (See relevant sections in the manuscript for metric-related caveats).
- Li et al. reported interannual fluctuations of the annual period between 363 and 367 days at ten CMONOC stations; if such period drift proves widespread, it challenges signal separation methods built on fixed-frequency assumptions.

**What are the implications of the main findings?**

- If the reported period drift of seasonal signals is confirmed on wider networks, future GNSS signal processing should move beyond fixed-frequency assumptions; time-varying frequency models (e.g., UKF_Amp_Period) offer a viable path forward.
- Multi-source fusion (GNSS + InSAR + GRACE) is essential for separating coupled geophysical signals that cannot be resolved by any single technique.
- Self-supervised foundation models (e.g., GNSS-FM, currently an unreviewed preprint) point to a possible paradigm shift in GNSS time series analysis, but their geographic fairness and physical interpretability remain unvalidated.

**Abstract:**

Precise signal extraction and geophysical interpretation of Global Navigation Satellite System (GNSS) coordinate time series constitute the core foundation for establishing the International Terrestrial Reference Frame (ITRF) and inverting surface mass redistribution. This paper reviews major advances in this field across four dimensions: model framework, signal characteristics, extraction methods, and geophysical mechanisms. Key findings include: (1) Maximum Likelihood Estimation (MLE) has become the recognized standard for linear trend extraction: by jointly estimating deformation parameters and the noise covariance, it corrects the up to 5–10-fold underestimation of velocity uncertainty that arises in conventional least-squares analyses when colored noise is present but a white-noise covariance is assumed; (2) in CMONOC benchmark tests reported by Wu et al., Variational Mode Decomposition (VMD) achieves an average 69.8% residual RMS reduction at 97.9% of stations—results that are promising but not yet independently replicated on other networks—while the self-supervised model GNSS-FM (currently an unreviewed preprint) represents an emerging intelligent analysis paradigm; (3) the combined effect of atmospheric, non-tidal ocean and hydrological loading explains about 42% of the residual power of the annual vertical signal globally after pole tide correction and reduces the weighted mean vertical annual amplitude from 4.19 mm to 3.19 mm, while for horizontal components the fraction explained by current loading models is much smaller (amplitude ratio, explained variance and RMS/WRMS reduction are distinct metrics and are not directly interchangeable). This paper further highlights that the annual period was reported to fluctuate between 363 and 367 days at the ten CMONOC stations analyzed by Li et al.—a time variability that, if general, challenges fixed-frequency signal separation methods, although apparent period changes may also arise from amplitude/phase modulation, spectral leakage, finite-record effects, colored noise or data gaps—and it identifies thermoelastic deformation (TED) as a long-neglected but potentially quantifiable component, based on a recently released preprint dataset that has not yet undergone peer review. Finally, key research prospects are outlined, including physics-informed fusion methods, self-supervised foundation models, and a unified multi-source inversion framework.

## 1. Introduction

**Figure 1:**
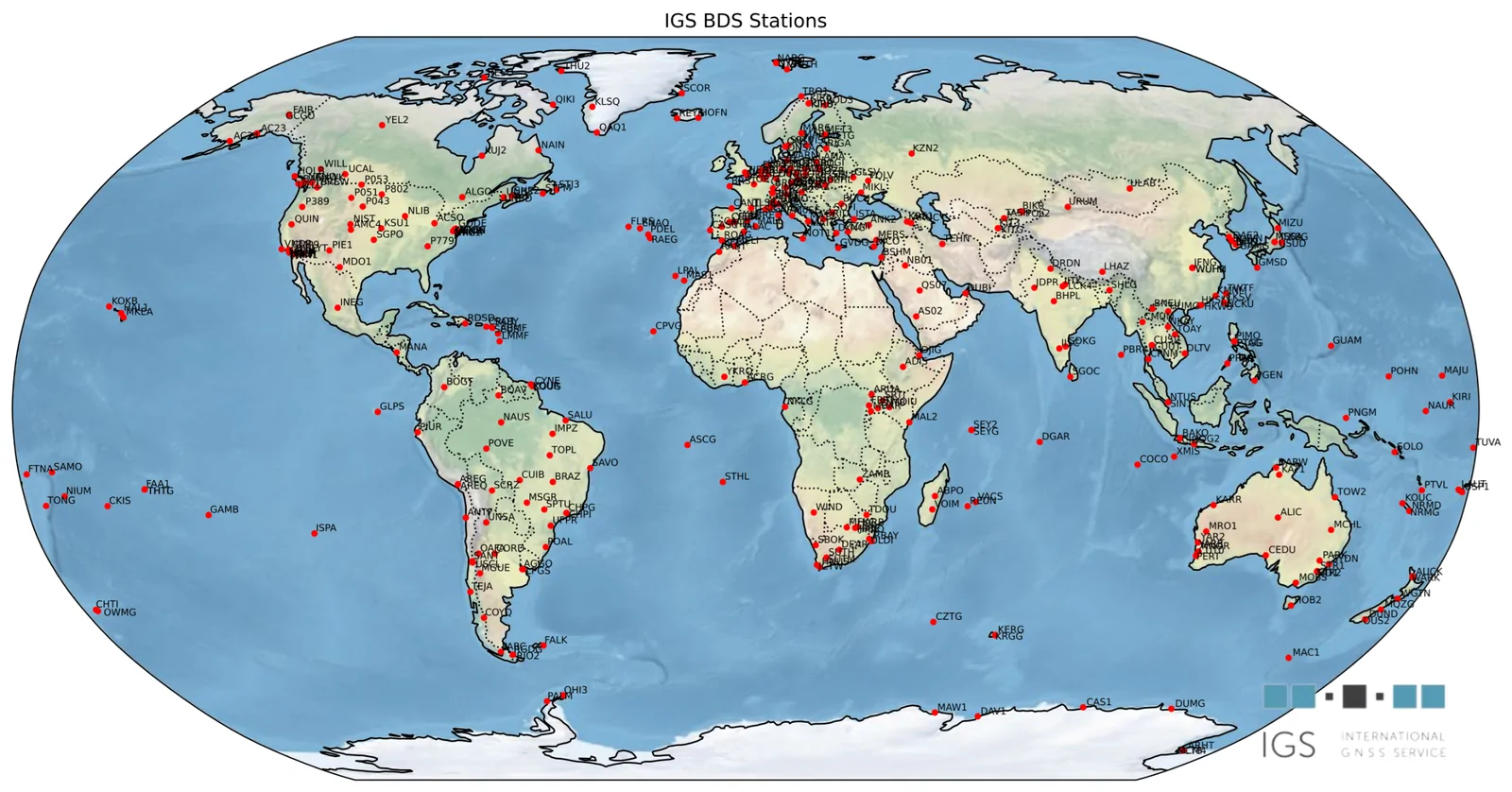
Global distribution of IGS tracking stations for multi-GNSS constellation. Map source: International GNSS Service (IGS) Real-Time Service. Retrieved from https://rts.igs.org/network-resources/#downloadable (accessed on 30 June 2026).

Global Navigation Satellite System (GNSS), with global coverage, all-weather operation and millimeter-level accuracy, has become an irreplaceable space geodetic tool for crustal deformation monitoring. Since the concept of Continuously Operating Reference Stations (CORS) was proposed in the 1990s, large-scale GNSS observation networks represented by the International GNSS Service (IGS) global tracking network and the Crustal Movement Observation Network of China (CMONOC) have been established successively [1]. The global distribution of IGS multi-GNSS stations is shown in Figure 1. With the coordinated development of multiple GNSS constellations, a new pattern of multi-constellation, multi-frequency integrated observations has taken shape worldwide.

GNSS coordinate time series serve as a critical bridge between space geodetic observations and geodynamic processes [1]. They embody the combined response of tectonic motion, surface mass redistribution, and observation system errors and can be decomposed into a superposition of deterministic signals and stochastic noise [2,3,4]. With over three decades of development, GNSS coordinate time series have become the core data source for establishing and maintaining the International Terrestrial Reference Frame (ITRF), constraining the patterns of crustal motion, and inverting surface mass redistribution [3] and play an irreplaceable role in characterizing millimeter-scale deformation fields in plate boundary zones and active tectonic regions [5].

GNSS coordinate time series show pronounced nonlinear deformation signals, primarily manifested as annual and semi-annual seasonal oscillations and long-term nonlinear trends. The vertical component typically has an amplitude of 4–10 mm, with amplitudes exceeding 10 mm at some inland stations, while the horizontal amplitude is roughly half that of the vertical component [6]. The physical origin of these nonlinear variations is the response of the elastic Earth to external loading: van Dam et al. [7] first demonstrated in 1994 that atmospheric pressure loading could account for up to 24% of the variance in GPS vertical coordinates; Dong et al. [6] quantified the combined contribution of atmospheric, non-tidal ocean, and hydrologic loading as approximately 42% of the residual power of the annual signal. In their latest systematic review, Li et al. [5] noted that significant discrepancies across studies in loading models, correction strategies, and evaluation metrics hinder the cross-regional generalization of quantitative conclusions. In addition, thermoelastic deformation [8] and regional anthropogenic activities (e.g., groundwater extraction [9], heavy precipitation [10]) are also important contributing factors.

Focused on the core goal of precise signal separation, researchers worldwide have developed a progressively evolving methodological system. Early least-squares harmonic fitting was built on the Gauss–Markov white-noise assumption; despite high computational efficiency, it ignores time-domain correlation. Williams [11] demonstrated that colored noise (especially flicker noise) can cause a 5–10-fold underestimation of velocity uncertainty, which directly motivated the development of the MLE method that enables simultaneous modeling of deformation parameters and noise covariance. To address time-varying periodic characteristics and nonlinear, non-stationary behaviors, adaptive methods including SSA, EMD, and VMD [12], which require no preset functional form, have emerged accordingly. From the perspective of physical mechanisms, the fusion of multi-source observations such as GNSS, GRACE, and InSAR is driving the transition from standalone deformation description to integrated interpretation of multi-field coupling [13,14].

Nevertheless, three core challenges remain in current research. First, environmental loading models explain only about 42% of the residual power of the annual vertical signal after pole tide correction, reducing the weighted mean vertical annual amplitude from 4.19 mm to 3.19 mm [6], while for the horizontal component the explained fraction is much smaller [15]. Second, environmental loading displacement products released by different institutions show significant discrepancies in amplitude and phase [16]. Third, the time variability of periodic signals is far more complex than previously recognized. Li et al. [17] reported that at the ten CMONOC stations they analyzed, the annual cycle fluctuates between 363 and 367 days; if confirmed on wider networks, this result challenges signal separation methods based on the fixed-frequency assumption.

Over the past three years, three key advances have delivered new analytical tools to tackle the above challenges. Li et al. [17] systematically demonstrated for the first time the theoretical and practical impacts of period offset and period variation on seasonal signal modeling. Teutschmann et al. [18] proposed GNSS-FM (currently an unreviewed arXiv preprint), the first self-supervised foundation model tailored for GNSS coordinate time series, representing a preliminary exploration of a possible paradigm shift from single-task dedicated models to general foundation models. Li et al. [5] presented a comprehensive systematic review of modeling methods for environmental loading effects.

This paper presents a comprehensive review organized around four dimensions: model framework, signal characteristics, extraction methods, and physical mechanisms. It compiles quantitative performance metrics reported in the literature for various methods and summarizes the pathways of multi-source data fusion and key future research directions.

With regard to scope and literature selection, this article is a narrative review rather than a formal systematic review. Relevant peer-reviewed publications (approximately 1994–2026) were surveyed through Web of Science, Scopus and Google Scholar using keywords such as “GNSS coordinate time series”, “seasonal signal”, “noise model”, “common mode error”, “transient deformation” and “environmental loading”. Priority was given to (i) large-sample studies on global or regional networks, (ii) quantitative method benchmark comparisons, and (iii) advances published within the last three years. The selection is intended to be representative rather than exhaustive, and no formal systematic-review screening protocol was applied.

## 2. Model Framework, Signal Composition, and Characteristics of GNSS Coordinate Time Series

As stated in the introduction, GNSS coordinate time series can be decomposed via a functional model into a superposition of deterministic signals and stochastic noise [3,4]. Of these components, deterministic time-varying signals are the direct manifestation of geophysical processes and observational events in the time series and represent the core target of signal extraction in time series analysis; stochastic noise is the superposition of observation system errors, random environmental disturbances, and weak unmodeled deterministic signals and serves as the main source of interference in signal extraction [19].

### 2.1. General Functional Model and Its Evolution

Since GNSS was first applied to crustal deformation and plate motion monitoring, the functional models of GNSS coordinate time series have undergone gradual iteration from simplified steady-state models to full-featured nonlinear models, driven by the continuous expansion of global observation networks and the accumulation of long time series observational data [2,4].

Early GNSS observation sequences had short time spans, and the core research objective was to estimate the long-term deformation rates of plate motion. A basic linear model consisting solely of a linear trend term, a periodic term and a stochastic term was widely adopted in the academic community [6], as expressed by:

(1) $$y(t)=a+vt+\sum_{k=1}^{n_{k}}A_{k}\left[\sin(\omega_{k}t+\varphi_{k})\right]+\varepsilon (t)$$

In this equation, a+vt denotes the linear trend term, which corresponds to long-term tectonic deformation such as plate motion; the periodic term corresponds to non-tectonic deformation driven by seasonal environmental factors; and ε(t) denotes the stochastic term, which comprises observation errors and unmodeled stochastic disturbances [2].

As the observation spans of global GNSS stations (e.g., IGS reference stations) have generally exceeded 20 years, features such as offsets and post-seismic nonlinear deformation in the time series have been widely documented. Two key factors drive this shift in understanding. First, during the realization of the ITRF, Altamimi et al. [3] found that failure to model post-seismic deformation would significantly impair the accuracy and stability of the global reference frame. Second, Gazeaux et al. [20] demonstrated via systematic step-detection experiments that unrecognized coordinate offsets severely distort velocity estimates.

Within their trajectory model framework, Bevis and Brown [2] proposed that GNSS coordinate time series can be expressed as a superposition of deterministic and stochastic models. The complete form of the general functional model widely adopted in the field is given by:

(2) $$y(t)=\sum_{i=0}^{n_{p}}p_{i}{\left(t-t_{R}\right)}^{i}+\sum_{j=1}^{n_{b}}b_{j}H(t-t_{j})+\sum_{k=1}^{n_{k}}A_{k}\sin(\omega_{k}t+\varphi_{k})+\sum_{l=1}^{n_{L}}a_{l}\log(1+\frac{t-t_{l}}{T_{l}})+\sum_{m=1}^{n_{M}}a_{m}(1-e^{-\frac{t-t_{m}}{T_{m}}})+\varepsilon (t)$$

Equation (2) comprises five components. The polynomial term $\sum_{i=0}^{n_{p}}p_{i}{\left(t-t_{R}\right)}^{i}$, which corresponds to long-term tectonic deformation, is generally taken as a first-order linear term, with $p_{0}=a$ and $p_{1}=v$ as self-explanatory coefficients. The step term $\sum_{j=1}^{n_{b}}b_{j}H(t-t_{j})$ models coordinate offsets induced by instrument replacement, co-seismic displacement and other events using the Heaviside step function. The periodic term $\sum_{k=1}^{n_{k}}A_{k}\sin(\omega_{k}t+\varphi_{k})$ corresponds to seasonal non-tectonic deformation, where $\omega_{1}=2\pi \;\mathrm{rad}/\mathrm{yr}$ denotes the angular frequency of the annual signal and $\omega_{2}=4\pi \;\mathrm{rad}/\mathrm{yr}$ that of the semi-annual signal. For the post-seismic deformation term, logarithmic and exponential functions are empirical trajectory functions. Following the ITRF2014 convention [3], logarithmic terms are commonly used to represent the long-tailed deformation often associated with fault afterslip, while exponential terms are commonly used for viscoelastic relaxation; this assignment is, however, not unique—both mechanisms can exhibit multi-timescale behavior and produce similar functional shapes—so function type alone cannot uniquely identify the underlying physical mechanism. ε(t) is the stochastic noise term, which can be expressed as white noise (WN), flicker noise (FN), random walk noise (RWN), or any combination thereof [4].

A key assumption implicit in Equation (2) deserves attention: the periodic term is formulated as sine-cosine functions with constant frequency. Li et al. [17] reported that at the ten CMONOC stations they analyzed, the annual cycle undergoes interannual variations ranging from 363 to 367 days; if such period drift is widespread, the fixed-frequency assumption will cause signal leakage into the noise component. The generality of this finding across other networks and the extent to which apparent period changes may instead reflect amplitude/phase modulation, spectral leakage, finite-record effects, colored noise or data gaps remain to be established. Furthermore, nonlinear coupling may exist between viscoelastic relaxation deformation and environmental loading deformation in post-seismic regions [21]. The Heaviside step function presumes instantaneous coordinate offsets and should therefore be applied with caution in high-frequency GNSS analysis.

These assumptions hold well for the vast majority of applications; however, explicit definition of their boundary conditions is critical for reliable analysis in extreme scenarios. In Equation (2), the polynomial term is generally taken as first-order, and annual and semi-annual components are the most dominant periodic signals; the model can accordingly be simplified as:

(3) $$y(t)=a+vt+\sum_{j=1}^{n_{b}}b_{j}H(t-t_{j})+\sum_{k=1}^{2}A_{k}\sin(\omega_{k}t+\varphi_{k})+\sum_{l=1}^{n_{L}}a_{l}\log(1+\frac{t-t_{l}}{T_{l}})+\sum_{m=1}^{n_{M}}a_{m}(1-e^{-\frac{t-t_{m}}{T_{m}}})+\varepsilon (t)$$

**Figure 2:**
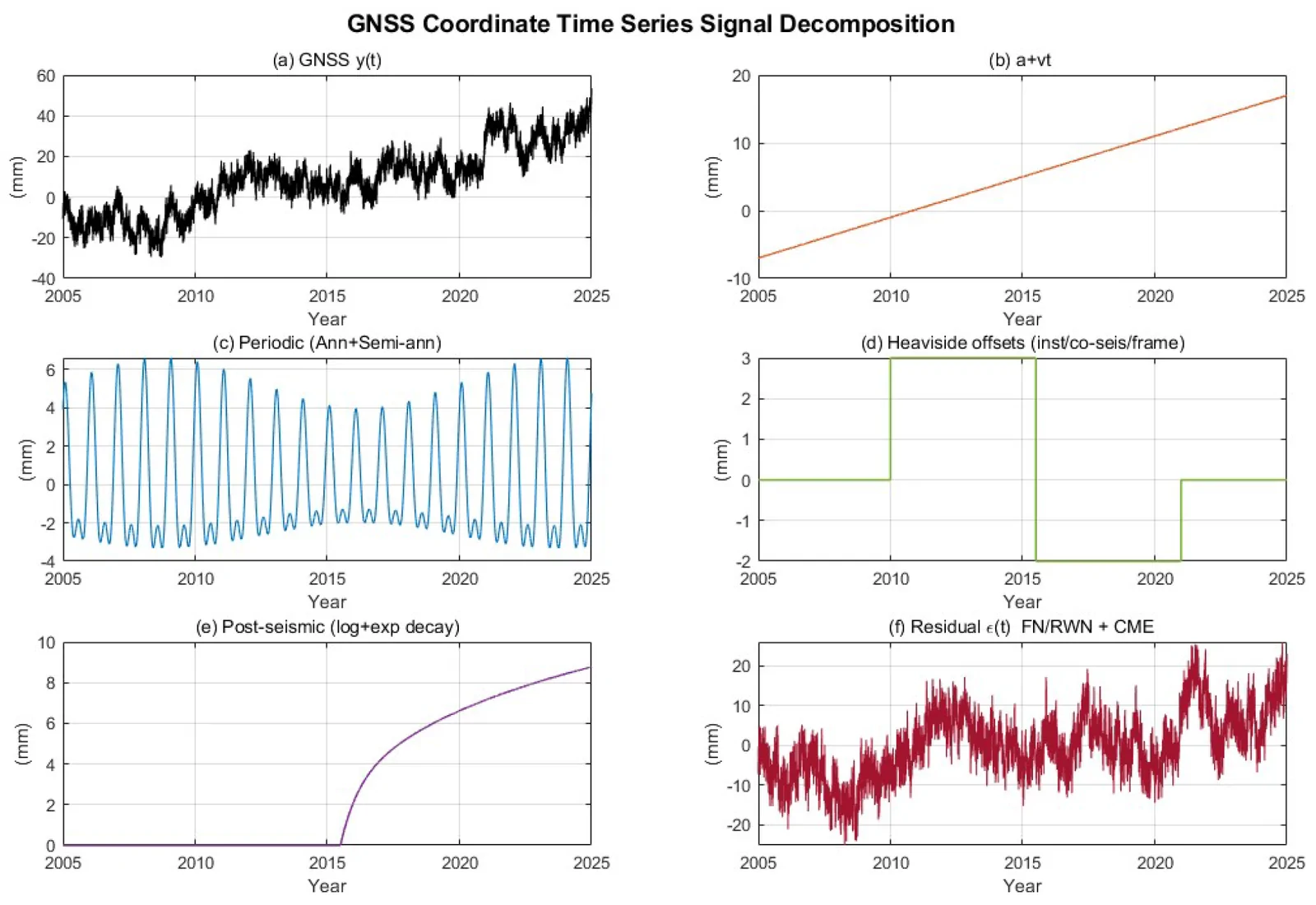
Schematic diagram of signal decomposition for GNSS coordinate time series.

Equation (3) clearly presents the five components of a GNSS coordinate time series, with a schematic decomposition of each component shown in Figure 2. The trend term primarily reflects long-term geodynamic processes such as plate tectonic movements. The periodic term is mainly driven by loading effects induced by the mass redistribution of surface fluids (atmosphere, oceans, and hydrologic systems) and thermal expansion effects. The step term generally arises from events such as earthquakes, instrument replacement, or antenna monument maintenance. The post-seismic deformation term serves to model the nonlinear relaxation process following strong earthquakes. The residual term comprises observation noise, common-mode errors, and unmodeled complex geophysical signals [22].

(Simulated data: time span 2005–2025; the linear trend has an intercept *a* = 5.0 mm and a velocity *v* = 1.2 mm/yr; the annual amplitude is 3.5 mm and the semi-annual amplitude is 1.8 mm, with amplitudes sinusoidally modulated by a 15-year cycle (modulation coefficient = 0.25); three-step offsets occur at 2010.0, 2015.5 and 2021.0, with magnitudes of +3.0, −5.0 and +2.0 mm, respectively; post-seismic deformation (starting at 2015.5) includes a logarithmic term 4.0ln(1 + Δ*t*/2.5) and an exponential term 2.5[1 − exp(−Δ*t*/0.6)], where Δ*t* is the post-seismic time in years; residual noise is the superposition of white noise (*σ* = 0.8 mm), flicker noise (*σ* = 1.2 mm), random walk noise (*σ* = 0.3 mm) and common-mode error (amplitude = 1.5 mm, containing annual, semi-annual and 0.5 cycles per year signals). References [2,5,10,21,23,24,25].)

For GNSS stations in seismogenic zones, their coordinate time series are susceptible to seismic effects, primarily manifested as co-seismic displacement, post-seismic velocity changes and nonlinear deformation [20]. ITRF2014 first developed post-seismic deformation (PSD) models for 123 GNSS stations worldwide with significant post-seismic deformation and released corresponding products [3]. For most stations not significantly affected by post-seismic deformation, the model can be further simplified as:

(4) $$y(t)=a+vt+\sum_{j=1}^{n_{b}}b_{j}H(t-t_{j})+\sum_{k=1}^{2}A_{k}\sin(\omega_{k}t+\varphi_{k})+\varepsilon (t)$$

A thorough understanding of the GNSS coordinate time series model framework serves as the cornerstone of subsequent precise signal separation, noise modeling and geophysical interpretation [1].

### 2.2. Deterministic Time-Varying Signals: Composition and Deformation Characteristics

Based on the general functional model, deterministic time-varying signals in GNSS coordinate time series can be decomposed into four core components according to their deformation characteristics, timescales and physical origins: linear trend term, periodic time-varying signals, transient/step-like time-varying signals, and long-term nonlinear time-varying signals [1,2]. The characteristics of each signal type directly determine the selection of subsequent extraction methods and the direction of geoscientific interpretation.

The linear trend term corresponds to the first-order polynomial term and serves as the core steady-state deformation signal in long-term GNSS time series, characterizing the average deformation rate of a station over the observation period [4]. Its key characteristics include long-term steadiness, constant linear velocity, low frequency, and absence of significant time variability. Horizontal linear trends are primarily driven by plate tectonic movements, while vertical trends arise from multiple factors including GIA, tectonic activity, and anthropogenic activities; the specific driving mechanisms are elaborated on in Section 4.1 [3,9,26].

Periodic time-varying signals correspond to harmonic fitting terms, with annual and semi-annual cycles as the dominant frequencies; their amplitudes and phases show pronounced time-varying characteristics [1]. Statistics from global GNSS stations show that most vertical annual amplitudes are below 10 mm (typically 4–10 mm), with a weighted mean amplitude of 5.47 mm before pole tide correction [6], and amplitudes can exceed 10 mm at some inland stations. The horizontal periodic signal amplitude is roughly half that of the vertical component.

Klos et al. [27] analyzed 26 IGS reference stations and found that the standard deviation of interannual variations in annual signal amplitude exceeds 0.5 mm at over 70% of stations, indicating that conventional fixed harmonic fitting can no longer meet precision requirements. The specific physical excitation sources (environmental loading, thermoelastic deformation, climatic modes, etc.) are elaborated on in Section 4.2.

Transient and step-like time-varying signals are non-stationary deformation signals in GNSS coordinate time series and are strongly associated with abrupt events. Step-like signals manifest as instantaneous coordinate jumps followed by permanent offsets, whereas transient deformation events typically last from days to years and exhibit large variability in deformation magnitude. Neither category follows a fixed periodicity, although some transient processes—slow slip events, episodic volcanic inflation and certain hydrological transients—recur quasi-periodically (see Section 4.3), so non-recurrence is not a defining property.

Step-like signals manifest as instantaneous coordinate jumps followed by permanent offsets. They arise primarily from co-seismic displacement, instrument replacement, antenna monument loosening, and reference frame jumps and can be modeled using the Heaviside step function.

Transient deformation signals present as short-lived nonlinear deformation processes (ranging from several days to several years), with typical forms including post-seismic afterslip, slow slip events, and deformation induced by volcanic magma migration. Typically, logarithmic functions are adopted to model the long-tailed decay commonly attributed to fault afterslip, exponential functions to characterize viscoelastic relaxation, and hyperbolic tangent functions to describe the evolution of slow slip processes [3,28]. These are empirical conventions rather than unique identifiers of the driving mechanism; distinguishing afterslip from viscoelastic relaxation requires spatial deformation patterns and physics-based forward modeling, not merely curve shape [29,30].

Long-term nonlinear time-varying signals have timescales ranging from months to decades and show nonlinear evolutionary patterns, differing significantly from steady-state linear trends and short-period oscillatory signals [1,2]. Based on their driving mechanisms, these signals fall into two broad categories: tectonically driven (post-seismic relaxation displacement) and non-tectonically driven (GIA, anthropogenically induced land subsidence, etc.). The specific causes and typical cases are elaborated on in Section 4.1 [3,20,26].

It should be noted that the above four categories form an operational classification tailored for method selection. In real coordinate time series, the boundaries between different signal types are ambiguous. Typical examples include the linear approximation treatment of GIA signals and the non-mass-loading-driven physical nature of TED. This awareness is critical for prudent interpretation of signal extraction outcomes (see Section 5 for detailed discussion).

### 2.3. Random Noise and Common-Mode Error

Stochastic noise corresponds to the residual term and acts as the dominant factor triggering biases in signal parameter estimation and the underestimation of deformation velocities [22]. Stochastic disturbances in GNSS coordinate time series are commonly characterized along two complementary dimensions: the temporal correlation structure (white noise versus temporally correlated colored noise such as flicker noise, random walk noise and Gauss–Markov noise) and the spatial coherence (station-independent errors versus spatially correlated common-mode errors). These dimensions are not mutually exclusive categories: the common-mode component may contain deterministic signals, such as large-scale environmental loading deformation or reference-frame artifacts, rather than purely stochastic noise (as further discussed below) [22,31,32].

Stochastic noise within GNSS coordinate time series consists of not only temporally uncorrelated white noise (WN) but also temporally correlated colored noise. Among colored noise components, flicker noise (FN) dominates, followed by random walk noise (RWN), alongside Gaussian–Markov noise and other minor noise types. Williams [11] systematically demonstrated that ignoring colored noise, particularly FN, underestimates the uncertainty of velocity estimates by a factor of 5–10.

Spatially correlated common-mode error (CME) is a systematic error prevalent in regional GNSS networks with strong spatial coherence, manifested as synchronous temporal variations in coordinate time series across most stations [33].

CME possesses three key features:

- High spatial correlation, which decays with inter-station distance [31,33];
- Multi-scale properties: global-scale contributions originate from ITRF realization errors, regional-scale components act as the dominant interference in network analysis, and local-scale coherent variations may arise from unmodelled environmental effects shared by closely spaced stations; strictly site-specific effects such as multipath or monument instability affect individual stations and should not be classified as common-mode errors unless spatial coherence across several stations is demonstrated [31,33];
- Broadband spectral characteristics, covering seasonal long-period to daily short-period bands, which lead to severe spectral aliasing with real geophysical signals [23,31].

The driving sources of CME fall into two categories: geophysical origins (large-scale environmental loading deformation) and data-processing systematic errors (reference frame biases, satellite orbit errors, atmospheric delay modeling errors, etc.) [34].

Recent research confirms that CME is not purely stochastic noise. Its low-frequency components contain authentic geophysical signals such as regional large-scale environmental loading deformation [22,31,32]. This shift in understanding provides fundamental guidance for developing CME filtering schemes.

## 3. Methods for Extracting Time-Varying Signals in GNSS Coordinate Time Series

The selection of signal extraction methods essentially boils down to matching prior knowledge of signal features with the inherent assumptions of each algorithm. Stationary linear signals are well suited to parametric approaches (e.g., LS, MLE). Non-stationary time-varying signals demand nonparametric algorithms that adaptively track signal evolution (e.g., SSA, VMD). High-dimensional nonlinearly coupled signals are increasingly processed via intelligent learning methods that can automatically fit complex mapping relationships (e.g., LSTM, Transformer, GNSS-FM).

However, such ideal one-to-one matching encounters two fundamental dilemmas in practical data processing. First, the intrinsic features of real signals remain unknown before analysis. It is impossible to judge whether a fluctuation belongs to stationary periodic components or time-variable periodic components and whether a trend is linear or nonlinear until the decomposition is completed. Second, multiple signal components coexist within a single time series, so there is no universal optimal algorithm. For example, MLE, which achieves optimal performance for linear trends, fails to separate time-varying periodic signals; meanwhile, VMD, effective for time-varying periodic signals, has low sensitivity to offsets.

Accordingly, when reviewing diverse extraction methods in this section, we not only elaborate their performance metrics but also emphasize the analysis of their applicable boundaries, i.e., the scenarios where each method yields reliable results and the conditions under which it may produce invalid outputs.

The previous chapter systematically elaborated on the binary decomposition framework of GNSS coordinate time series, consisting of deterministic signals and stochastic noise, as well as the deformation characteristics of the four categories of deterministic signals. Centered on the core objectives of accurate signal-noise separation and effective decoupling of multi-source coupled signals, this section sequentially reviews the fundamental principles, applicable scenarios and performance characteristics of matched extraction methods according to the signal classification system established above. Quantitative performance metrics derived from large-sample observational datasets are provided for each method, enabling readers to form quantitative judgments on the merits and drawbacks of different algorithms. Table 1 summarizes the comprehensive comparison of all aforementioned methods.

Table 1 conducts vertical comparisons grouped by algorithm category. To help readers rapidly screen suitable techniques for practical needs, Table 2 presents method recommendations sorted by application scenarios across two dimensions: signal category and research emphasis. The lookup workflow follows two steps: determine the target signal type (table row), clarify your research priority (table column), and refer to the intersecting cell for the suggested algorithm.

### 3.1. Linear Trend Component Extraction Methods

Linear trend components feature long-duration stationary behavior and constant linear velocity. The extraction goal is to reliably estimate station deformation velocities and corresponding uncertainty bounds [1,11].

The LS linear fitting method serves as the benchmark technique to extract stationary GNSS deformation signals and forms the mathematical backbone of trend estimation in the standardized workflows of IGS analysis centers and ITRF realization. It should be noted that operational ITRF combination goes far beyond ordinary least squares under an independent white-noise assumption, employing weighted stacking, variance component estimation and rigorous outlier handling [3]; the classical single-station model discussed below adopts the Gauss–Markov white-noise assumption for mathematical tractability. Built upon this assumption, the method establishes a stationary deformation fitting model via linear regression, featuring a concise model structure and clear physical interpretability for solved parameters [1,11]. Williams [11] systematically demonstrated that when GNSS time series contain temporally correlated (colored) noise—flicker noise in particular—but the uncertainty analysis is performed under a white-noise assumption, the uncertainty of velocity estimates can be underestimated by up to a factor of 5–10, with the exact magnitude depending on the noise spectrum, sampling interval, record length, data gaps and offsets. If the residuals are genuinely white, independent and correctly modeled, the conventional least-squares uncertainty estimates remain statistically valid; the bias is therefore a consequence of stochastic-model misspecification rather than of least squares itself. Such underestimation becomes drastic when flicker noise dominates the dataset, undermining the credibility of plate motion velocity inversion and subsequent geophysical interpretation.

MLE with colored noise constraints fundamentally addresses the issue of underestimated uncertainty inherent to conventional LS, as it jointly estimates deformation signal parameters and noise model parameters. It has emerged as the widely accepted standard algorithm for linear trend extraction and stochastic noise modeling of GNSS time series [36].

In its most widely used GNSS formulation, MLE models the total noise covariance as a linear combination of white and colored noise components and jointly estimates deformation and noise parameters by maximizing the log-likelihood function. We emphasize that this is a choice of covariance model rather than an inherent constraint of MLE: the likelihood framework is equally applicable to power-law noise, autoregressive, state-space, heteroscedastic or non-Gaussian covariance structures [36,37,38].

Statistics from global GNSS monitoring stations indicate that the white noise plus flicker noise (WN + FN) model is the optimal noise combination for most long-duration time series (>3 years) [36]. In contrast, the WN + random walk noise (RWN) model or other more complex noise schemes remain viable alternatives for a subset of stations [37].

Noise model selection generally relies on the Akaike Information Criterion (AIC) and Bayesian Information Criterion (BIC). BIC imposes harsher penalties on model parameters, so it favors more parsimonious stochastic noise models for long GNSS time series [36,37].

Sun et al. [39] further systematically quantified the coupled impacts of time series span and data gaps and reported two related but distinct thresholds. First, for datasets shorter than about 8 years, sophisticated noise models incorporating flicker noise and random walk noise (FNRWWN, FNWN) are easily misidentified as the simpler Gaussian–Markov noise model (GGMWN), i.e., short records cannot reliably resolve colored noise components. Second, the same study estimated that roughly 12 years of observations are required before a reliable stochastic noise model can be established at all. These two thresholds address different aspects of the same identifiability problem—component separation versus overall model reliability—and together offer quantitative constraints on the minimum data standards for MLE-driven stochastic noise modeling.

MLE can effectively compensate for the underestimation of velocity uncertainty yielded by LS [36,37] and accommodates unevenly sampled time series with data gaps. Nevertheless, MLE suffers from three inherent drawbacks.

a. Computational overhead limitation

MLE requires inversion of an n×n covariance matrix, bringing high computational complexity of $O\left(n^{3}\right)$. For ultra-long time series with tens of thousands of observation epochs, the computational load becomes a significant bottleneck. Cucci et al. [38] proposed the Generalized Wavelet Moment Method (GMWMX) within a semiparametric framework, which cuts the computational complexity down to nearly linear complexity. This algorithm enables batch MLE processing for large-scale GNSS networks. Still, extensive verification is needed to confirm whether its statistical efficiency matches that of full MLE under complicated stochastic noise conditions.

b. Sensitivity to model specification

The optimal statistical performance of MLE relies entirely on accurate noise model specification. If the real stochastic noise structure deviates from the predefined noise combination, the resulting parameter estimates will lose optimality.

c. Stationarity prerequisite

MLE operates under the assumption that deformation signals are stationary. This assumption introduces estimation biases when processing time-varying periodic signals [17,27] and undetected step offsets [20].

### 3.2. Methods for Extracting Periodic Time-Varying Components

Conventional modeling of GNSS periodic components rests on an implicit but highly influential assumption: all signal frequency constituents are predefined and invariant, with a fixed annual cycle of 365.25 days and semi-annual cycle of 182.625 days. This postulate has been extensively adopted ever since the landmark study conducted by Dong et al. [6].

However, Li et al. [17] provided the first systematic examination of this assumption from both theoretical and practical dimensions. Using observational datasets from 10 CMONOC stations, they reported two critical features of seasonal cycles:

- Period offset: The actual annual cycle fluctuates between 363 and 367 days, departing from the conventional fixed-period hypothesis by up to ±2–3 days;
- Period variation: Such offsets do not arise from stochastic noise but follow a systematic interannual drift pattern.

Two caveats are in order. First, this result was obtained from ten CMONOC stations; its representativeness for other regional and global networks requires further verification. Second, apparent period variations can also be induced by amplitude and phase modulation, spectral leakage, finite-record effects, colored noise, data gaps, or the superposition of multiple geophysical components, so period-drift estimates should be interpreted together with these confounding factors. Consequently, the implication for fixed-frequency methods should be regarded as a potential systematic bias that merits dedicated comparative testing, rather than a universally demonstrated failure mode.

The Unscented Kalman Filter model for time-varying amplitude and period (UKF_Amp_Period), which jointly estimates temporally varying amplitudes and cycle lengths, boosts the fitting precision of annual and semi-annual components by 61% relative to conventional WLS and 40.6% relative to SSA.

If the reported period drift is confirmed on wider networks, the methodological implication would go beyond mere performance gains of a single algorithm: signal decomposition techniques built upon fixed-frequency priors, including parametric harmonic fitting as well as nonparametric SSA and VMD, may generate systematic biases when processing time series with significant period offsets—the magnitude of which has not yet been quantified through comparative testing.

Accordingly, the detection of period shifts is not simply an additional correction term appended to GNSS periodic modeling workflows; instead, it triggers a thorough re-evaluation of the field’s foundational assumptions. The nonstationarity embedded within GNSS seasonal components manifests not only in time-varying amplitude and phase but also in temporally variable frequency itself.

GNSS periodic components are dominated by annual and semi-annual frequency constituents, with marked temporal variability in amplitude and phase that renders them inherently nonlinear and non-stationary (see Section 4.2 for details). Classical LS and MLE algorithms are only capable of fitting stationary periodic signals with constant amplitudes and phases and will yield systematic biases when deployed to process time-varying periodic components.

SSA decomposes the trajectory matrix of a GNSS time series via singular value decomposition (SVD), separating the original sequence into trend, periodic and stochastic noise components. It adaptively extracts temporally varying periodic components with no prior specification of signal frequencies or functional forms [25].

Large-sample experiments using CMONOC station datasets demonstrate that SSA achieves substantial RMS residual reduction for 90.5% of stations [24]. In terms of signal extraction capability, SSA outperforms conventional fixed-frequency harmonic fitting but performs slightly worse than VMD (see the comparative analysis at the end of this section). Its primary drawback is that decomposition outputs are highly sensitive to the selected window length L. To date, no universal criterion exists for optimal parameter tuning. Furthermore, SSA shows poor adaptability to ultra-short time series and datasets with significant step offsets.

Proposed by Huang et al. [40], Empirical Mode Decomposition (EMD) adaptively decomposes a time series into a set of intrinsic mode functions (IMFs) via a data-driven sifting process. EMD outperforms conventional algorithms for processing nonlinear and non-stationary time series, yet the original EMD suffers severe mode mixing and end effects. Subsequent improved variants, namely Ensemble Empirical Mode Decomposition (EEMD) and Complementary Ensemble Empirical Mode Decomposition with Adaptive Noise (CEEMDAN), mitigate these inherent defects progressively through noise-assisted ensemble strategies [41]. For GNSS time series applications, Qiu et al. [41] introduced the EMD framework to GNSS coordinate time series processing, substantially boosting the precision of missing epoch gap filling and nonlinear component extraction.

VMD converts signal decomposition into a constrained variational optimization problem, which achieves separate extraction of each modal component via adaptive segmentation in the frequency domain [42].

Distinct from EMD’s recursive sifting scheme, VMD adopts a non-recursive decomposition framework and restricts each decomposed mode to a Band-Limited Intrinsic Mode Function (BLIMF), fundamentally eliminating mode mixing from a theoretical perspective. Wu et al. [12] conducted large-sample experiments using CMONOC station datasets. Their results show that VMD yields positive RMS residual improvements at 97.9% of all stations. For vertical coordinate components, the average RMS improvement ratio reaches 69.8%, with a peak value up to 104.6%. Accordingly, within the CMONOC benchmark reported by Wu et al. [12], VMD ranks among the effective algorithms documented for non-stationary periodic component extraction. Three qualifications apply: (i) these benchmark results originate from our prior work and have not yet been independently replicated on other geodetic networks; (ii) residual RMS reduction quantifies residual suppression rather than guaranteed signal recovery—an algorithm may lower residual RMS through overfitting or by transferring genuine geophysical signal into (or out of) extracted modes; and (iii) as further discussed in Section 5, VMD’s fixed central frequency bands may absorb genuinely period-drifted seasonal signals into the residual, so part of the RMS improvement may reflect the metric itself rather than superior signal separation. The primary drawback of VMD is that decomposition outputs are extremely sensitive to the pre-specified mode count K, and no universal automatic criterion has been established to determine the optimal value of K.

A systematic comparison of SSA, EMD, and VMD across three dimensions is warranted.

From the perspective of prior signal feature information: If the dominant frequency of quasi-periodic components is roughly known, VMD can efficiently isolate narrowband modes by virtue of its frequency-domain constraints. When spectral signatures remain entirely unknown, SSA’s fully data-driven architecture becomes preferable. For signals with severe nonlinearity and nonstationarity (e.g., post-seismic time series), the fully localized decomposition scheme of EMD-family algorithms stands out as the optimal choice, albeit at the cost of potential mode mixing artifacts. Using CMONOC stations as a unified benchmark, Wu et al. [12,24] reported positive RMS residual reduction at 97.9% of stations for VMD (average improvement of 69.8%), compared with SSA’s 90.5% success rate; both figures derive from the same research group and await independent replication.

In terms of computational overhead: SSA is the fastest computation and fits rapid batch processing workflows for large-scale GNSS networks. VMD relies on iterative optimization, so it is more applicable to refined analysis of individual stations or small networks where precision is the top priority.

**Figure 3:**
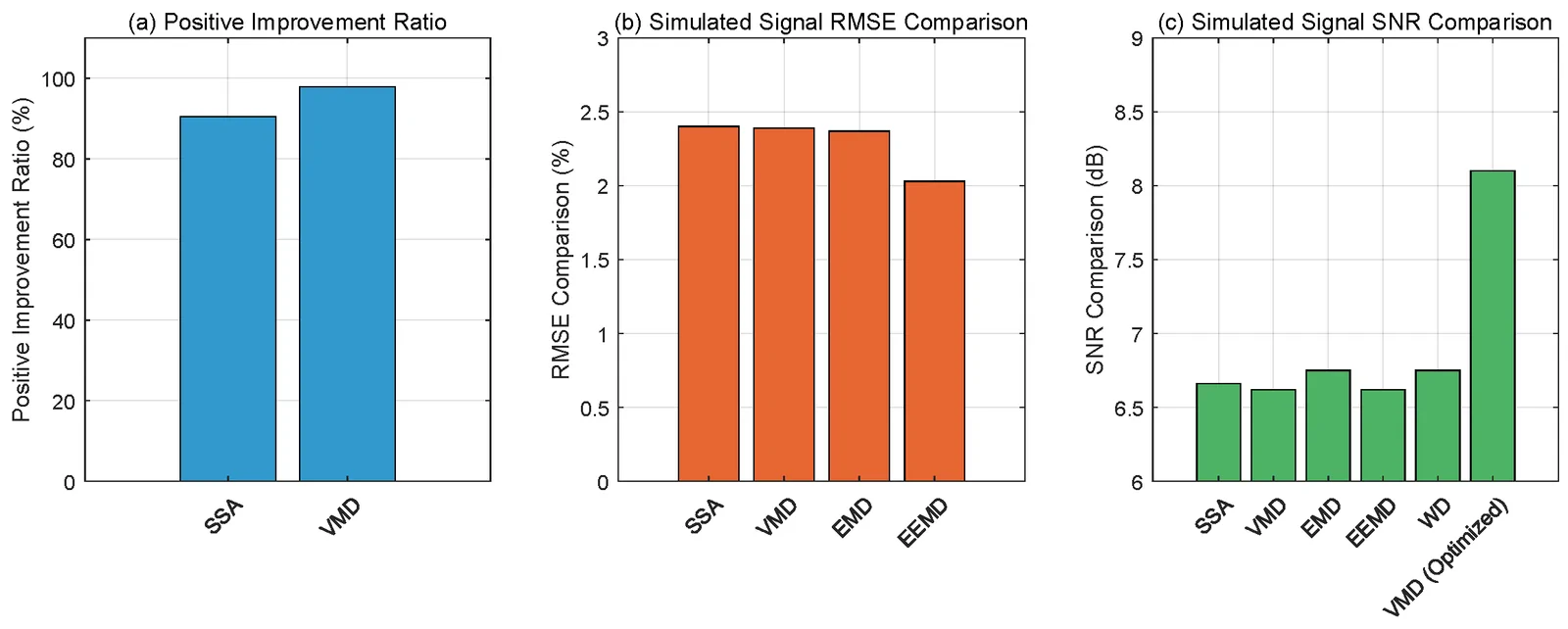
Performance comparison of various nonparametric algorithms for extracting time-varying GNSS components. VMD achieves positive residual RMS improvement at 97.9% of CMONOC stations (average 69.8%), outperforming both SSA and EMD. Data sources: Wu et al. [12], Wu et al. [24]. These benchmark results originate from the authors’ prior work on the CMONOC network only; independent replication on other networks is pending, and residual RMS improvement does not by itself guarantee complete signal recovery (see Section 5).

Regarding physical interpretability: VMD’s band-limited modal restrictions yield well-defined narrowband frequency signatures, giving it the highest interpretability among the three methods. SSA’s eigenvalue spectrum offers a reference threshold for separating signal components from stochastic noise, but the physical connotation of each reconstructed component (RC) can only be clarified via post hoc analysis. The intrinsic mode functions (IMFs) decomposed by EMD-family approaches generally lack explicit physical correspondence to geophysical signals. Figure 3 illustrates the disparities in technical frameworks across these methods.

To tackle three practical bottlenecks encountered by the above nonparametric decomposition algorithms in real-world processing workflows, recent studies have put forward targeted improvements from distinct technical perspectives:

a. Mitigation of parameter sensitivity.

Conventional EEMD is highly susceptible to two manually preset hyperparameters: the amplitude of added white noise and the ensemble averaging count. Li & Guo [43] developed an adaptive EEMD scheme that dynamically adjusts the noise injection strategy and tunes noise amplitude adaptively according to the signal features of the ongoing decomposition stage. This approach drastically reduces reliance on predefined parameter values. However, further cross-regional validation is still required to verify whether its self-adaptive tuning rules maintain universal applicability across diverse geodetic networks.

b. Processing gappy time series.

Data gaps are ubiquitous within GNSS coordinate time series, triggered by instrument malfunctions, communication outages and other operational faults. Ji et al. [44] proposed the Efficient Improved Singular Spectrum Analysis (ISSA), which integrates an iterative interpolation framework and an adaptive window-selection mechanism. It retains stable extraction performance for periodic components even when the data missing ratio reaches 30%. A critical caveat is that ISSA’s iterative interpolation relies intrinsically on SSA itself to reconstruct missing epochs. If gaps follow a nonrandom distribution (e.g., continuous missing segments concentrated within a single seasonal window), interpolated values will be biased toward the seasonal signatures of that timeframe, introducing systematic biases to subsequent trend and periodic component estimation.

c. Elimination of multivariate eigenvector mixing.

When processing regional GNSS networks, classic Multi-channel Singular Spectrum Analysis (MSSA) suffers from eigenvector mixing across different stations, which degrades the physical separability of decomposed modes. To resolve this issue, Fu et al. [45] presented Varimax-rotated MSSA, which incorporates Varimax orthogonal rotation (a standard factor-analysis technique that boosts factor interpretability) to enhance the separation degree of multi-channel signal constituents. However, Varimax rotation operates under the maximized inter-mode variance criterion; this assumption may cause faint geophysical signals to be overwhelmed by high-power dominant modes when the amplitude disparity between signal components is substantial.

### 3.3. Methods for Extracting Transient and Step Offset Components

Step offsets correspond to permanent instantaneous displacements, including co-seismic offsets induced by earthquakes and coordinate jumps caused by instrument reconfiguration. In contrast, transient deformation components refer to short-lived non-permanent geodetic motions such as post-seismic afterslip and slow slip events [20]. The core extraction target for both categories of components is to precisely pinpoint the timing of abrupt shifts and quantify the magnitude of associated deformation.

For cases with known event timings, event-driven parametric fitting frameworks are adopted. Specifically, the Heaviside step function models permanent step offsets; following the ITRF2014 convention [3], logarithmic formulations are commonly used for the long-tailed decay typically attributed to fault afterslip, and exponential functions for viscoelastic relaxation—with the caveat that neither function type uniquely identifies the physical mechanism [3,20]; and the hyperbolic tangent function describes the full evolutionary cycle of slow slip events [28].

ITRF2014 has incorporated post-seismic deformation (PSD) models constructed from logarithmic and exponential terms [3], which thoroughly verifies the practical applicability of such parametric techniques.

Parametric fitting algorithms offer clear physical interpretability and precise quantification of deformation magnitudes. Nevertheless, they hinge entirely on prior event information and fail to detect unrecognized transient components.

Yang & Xu [46] proposed an Adaptive Kalman Filter (AKF) built upon the Sage sliding window weighted sum algorithm and variance component estimation. This method continuously estimates and refreshes the covariance matrices of process noise and measurement noise in real time, substantially boosting the filter’s robustness under dynamic geodetic conditions.

Its key strength lies in real-time dynamic parameter estimation, rendering it a core technique for high-rate GNSS deformation monitoring and geological hazard early warning.

Extended Kalman Filter (EKF) and Unscented Kalman Filter (UKF) are further generalized to accommodate nonlinear state-space systems. Still, both approaches demand a predefined structural form of the state model.

For transient components with fully unknown and unconstrained morphological features, nonparametric decomposition algorithms including SSA and VMD can adaptively detect abrupt shifts within time series. Nonetheless, decomposed modes lack clear physical interpretability, and their deformation quantification precision is generally inferior to parametric and semiparametric schemes [42,47].

In the field of refined detection of transient components, methodological advances achieved in the past three years fall into three progressive hierarchical levels: precise extraction of deformation time series for recorded events, systematic screening for unrecognized events within fixed regional networks, and real-time unsupervised intelligent detection:

a. Refined extraction of deformation time series for recorded geophysical events.

Using the 2014 Ludian M6.5 earthquake as a case study, Song et al. [21] adopted a joint scheme integrating Kalman Filtering, the first-order Gaussian–Markov stochastic noise model and PCA. They successfully resolved the two-stage evolutionary pattern of post-seismic transient components. Continuous deformation governed by fault afterslip prevailed from 2014 to early 2016, followed by a recovery stage dominated by viscoelastic relaxation starting in early 2016.

This joint technique verifies the powerful capacity of parametric filtering frameworks to separate multi-stage post-seismic deformation signals. However, its mandatory dependence on prior seismic event constraints means the algorithm is optimized for validating documented events rather than detecting unforeseen transient offsets.

b. Systematic screening of undocumented transient offsets within monitored regional networks.

Proposed by Shi et al. [48], Multi-station Hankel Spectrum Analysis (MHSA) jointly constructs a Hankel matrix from multi-site time series and conducts singular value decomposition (SVD) to achieve precise separation of transient components across spatial and temporal domains. Using GNSS datasets spanning 2006 to 2022 at Akutan Volcano, this algorithm successfully captured the recurrent periodic pattern of exponential magmatic inflation events.

Joint multi-station processing under MHSA greatly elevates the signal-to-noise ratio of weak transient components. At the same time, its computational burden surges sharply with the growth of station quantities, leaving its processing efficiency for large-scale GNSS geodetic networks requiring further optimization.

c. Real-time unsupervised intelligent detection of transient offsets.

Tanaka et al. [49] were the first to apply machine learning model training directly to real GNSS coordinate time series, attaining a single-site slow slip event (SSE) detection accuracy of roughly 75% across southwest Japan.

The core innovation of this work lies in training on actual observational data instead of synthetic datasets, which substantially mitigates the weak generalization that affects models trained purely on simulated signals.

Even so, the 75% single-site detection accuracy leaves 25% of SSEs subject to omission or false classification. Moreover, the model’s hyperparameters optimized for southwest Japan show strong specificity toward subduction-zone SSEs. Additional cross-setting transfer learning validations are required to confirm its transferability to strike-slip fault environments [50].

### 3.4. Methods for Extracting Nonlinear Long-Term Time-Varying Components

Nonlinear long-term time-varying components are characterized by long periodicity, inherent nonlinear nonstationarity and unconstrained morphological patterns. Their core extraction objective is to separate nonlinear trend signals from periodic constituents and stochastic noise [1,2].

For slowly varying long-term geodetic deformations with weak nonlinearity, smoothing splines and compensated least-squares semiparametric regression serve as mainstream processing tools.

Smoothing splines introduce a penalty term to strike a balance between fitting precision and model smoothness, integrating the high computational efficiency of parametric algorithms and the strong nonlinear adaptability of nonparametric counterparts.

The compensated least-squares model partitions time series into parametric constituents and nonparametric nonlinear deformation terms and conducts joint estimation via a regularization matrix. It enables adaptive fitting of weak nonlinear deformation while retaining clear physical interpretability.

Both approaches share identical drawbacks: the tuning of regularization hyperparameters relies heavily on empirical judgment, and they are only valid for scenarios featuring mild nonlinearity.

For long-term trends with strong nonlinearity and nonstationarity, such as post-seismic deformation and nonlinear uplift driven by glacial isostatic adjustment (GIA), SSA and VMD adaptively isolate nonlinear long-term components by retaining the top one or two low-frequency modes with dominant energy ratios.

VMD fundamentally mitigates the mode mixing inherent to SSA and achieves better decomposition performance when processing strongly nonlinear ground subsidence datasets [12,24].

### 3.5. Approaches for Suppressing Stochastic Noise and Common-Mode Errors

Stochastic noise embedded in GNSS coordinate time series falls into two categories: time-uncorrelated white noise and temporally correlated colored noise, including flicker noise (FN) and random walk noise (RWN) [11,36].

Maximum Likelihood Estimation (MLE) quantifies the amplitude of each noise component and revises the uncertainty of geodetic velocity, correcting substantial velocity-uncertainty underestimation caused by ignoring temporally correlated noise; this has become the internationally recognized standard noise modeling workflow [11,36]. This scheme performs optimally for long-duration time series spanning more than three years; when observational records are shorter than two years, its capacity to separate FN and RWN degrades markedly.

From the perspective of signal decomposition, Yang et al. [51] processed datasets from 226 CMONOC stations. Their results show that two joint denoising schemes, namely CEEMD coupled with wavelet denoising (CEEMD&WD) and VMD combined with WD, reduce flicker noise amplitude from 19.90 mm/yr^0.25^ to 2.8 mm/yr^0.25^, corresponding to an 86% attenuation rate. Beyond temporal noise models, accounting for physical correlations in stochastic models has likewise been shown to be essential for robust GNSS parameter estimation under satellite-constrained conditions [52], reinforcing the general principle that covariance misspecification degrades geodetic estimates.

The two joint approaches outperform standalone CEEMD (6.84% noise reduction) and standalone VMD (16.88% noise reduction), respectively, demonstrating the clear advantage of multi-algorithm collaborative denoising over single-component decomposition.

Spatially correlated common-mode errors (CMEs) constitute pervasive systematic biases within regional GNSS geodetic networks, manifesting as synchronous time-varying signatures across most stations [33].

Principal Component Analysis (PCA) and Empirical Orthogonal Function (EOF) decomposition represent the most widely adopted spatial filtering schemes for CME mitigation, which were formally introduced into GNSS coordinate time series analysis by Dong et al. [33].

To address the critical vulnerability of conventional PCA to outliers, researchers have developed enhanced variants including iterative PCA and Robust PCA (RPCA). Such improved algorithms cut the RMS of coordinate residuals in the East, North and Up (E, N, U) components by 51.86%, 59.09% and 57.42%, respectively [35].

Independent Component Analysis (ICA) implements blind source separation by maximizing signal non-Gaussianity. Unlike PCA, it provides more effective separation of spatially correlated common-mode errors (CMEs) from large-scale genuine tectonic deformation components.

Taking the GNSS network covering the Antarctic Peninsula as the testbed, Li et al. [35] verified that regional filtering based on ICA cuts the RMS of East, North and Up (E, N, U) coordinate residuals by 44.69%, 26.94% and 34.87%, respectively, which represents a significant improvement relative to conventional PCA.

Feng et al. [32] proposed a modified ICA approach specifically designed for incomplete GNSS coordinate time series. By leveraging the reversible properties between the original time series and principal components to recover missing data prior to FastICA processing, their method was validated on 24 stations in the North China Network (2011–2019). The modified ICA captures approximately 17–18% more signal energy per component than standard iterative ICA, and validation through random data deletion confirms that the reconstruction error remains consistently lower even when a substantial fraction of observations is missing, demonstrating its robustness for regional CME extraction in practical networks with data gaps.

Multi-channel Singular Spectrum Analysis (MSSA) exploits spatiotemporal correlations among multi-site time series, showing far stronger capability than single-channel SSA in extracting faint regional common deformation components [23,53].

Proposed by Wu [4], the Phase-Constrained Annual Clustering Algorithm (PACA) adopts sub-cluster spatial filtering to greatly improve the coherence of annual displacement constituents within individual clusters. This overcomes the key limitation of conventional global PCA, which frequently misclassifies genuine regional tectonic signals as common-mode errors (CMEs).

In terms of upgraded CME filtering techniques, Li et al. [54] proposed a variational Bayesian PCA (VBPCA) approach to estimate and extract CME from incomplete GNSS position time series. The method naturally handles missing data within the Bayesian framework and employs a variational expectation-maximization iterative algorithm to identify the principal subspace. Tested on 44 continuous GNSS stations in Southern California, VBPCA automatically selects the optimal number of principal components and avoids overfitting, achieving lower CME relative errors than traditional PCA-based methods when more missing data is present. Its key advantage lies in the principled treatment of data gaps, rather than requiring complete records or ad hoc interpolation; the probabilistic model simultaneously estimates the missing values and the common spatial patterns, making it particularly suitable for regional networks where station outages are frequent.

### 3.6. Machine Learning and Intelligent Time Series Decomposition Methods

The analysis of GNSS coordinate time series is confronted with a fundamental epistemological challenge: residual geodetic constituents stemming from unidentifiable physical sources persist within time series, which prevents the construction of explicit functional models for their separation and creates a unique application niche for machine learning algorithms.

Conventional parametric approaches such as LS and MLE presuppose that all deformation constituents can be expressed via predefined functional forms. Nonparametric decomposition schemes including SSA and VMD relax this constraint but still rely on strict mathematical premises, such as narrowband assumptions and orthogonality constraints.

Machine learning opens an alternative paradigm: it automatically learns the implicit mapping between deformation constituents and stochastic noise from massive observational datasets without requiring predefined functional forms for geodetic signals.

The appeal of this data-driven framework lies in its unique capacity to capture statistical signatures of subtle geodetic variations that remain unexplainable under existing physical theories, for instance, the driving origins of horizontal periodic constituents. Even when the underlying physical mechanisms stay ambiguous, machine learning can retrieve such latent deformation patterns purely from data statistics.

Gaussian Process Regression (GPR) constructs the covariance matrix of time series via kernel functions to implement Bayesian nonparametric fitting for nonlinear geodetic constituents. It provides both fitted deformation outputs and corresponding uncertainty estimates and has been widely adopted for extracting nonlinear long-term deformation components and filling data gaps within GNSS records.

Recurrent Neural Network (RNN) architectures and their upgraded variants, Long Short-Term Memory (LSTM) and Gated Recurrent Unit (GRU), efficiently capture short-timescale temporal dependencies embedded in time series, producing reliable performance for GNSS nonlinear fitting and missing data imputation.

Benefiting from the self-attention mechanism, Transformer architectures support global feature extraction from lengthy GNSS time series, showing strong capability in joint spatiotemporal decomposition of multi-site deformation signals.

Furthermore, Graph Neural Networks (GNNs) exploit spatial topological connections between stations to perform collaborative denoising and possess distinctive strengths for modeling multi-station GNSS spatiotemporal datasets [55].

To resolve the “black-box” limitation inherent to deep learning architectures, Physics-Informed Neural Networks (PINNs) embed physical governing equations, including elastic loading theory and elastic mechanics formulations, as soft constraints within the loss function. This framework enables seamless integration of data-driven learning and physical prior knowledge.

In a review focusing on joint seismic-electromagnetic inversion, Ma et al. [56] concluded that physics-constrained learning schemes are capable of improving the physical consistency of geodetic parameter inversion. Such methodology provides meaningful reference for GNSS coordinate time series processing.

Within GNSS time series research, preliminary investigations have embedded elastic load Green’s functions [57] and viscoelastic relaxation constitutive equations as regularization constraints for neural networks. Idealized numerical tests on partial load deformation modeling and post-seismic deformation forecasting have validated the latent merits of introducing physical governing relations.

Even so, PINNs encounter two critical bottlenecks when deployed on real GNSS datasets. First, no adaptive tuning mechanism exists to balance the weight of physical constraint terms and data fitting residuals. Second, multi-objective optimization struggles to guarantee stable convergence amid non-convex loss landscapes. Accordingly, its claimed strong generalization under limited sample sizes demands systematic validation using field GNSS observations.

Overall, the core strength of machine learning algorithms lies in their capacity to automatically capture intricate nonlinear geodetic signatures, delivering higher decomposition accuracy than conventional approaches under large-sample conditions.

Their prominent drawbacks consist of three aspects: heavy reliance on abundant high-quality labeled datasets, poor model interpretability, and unvalidated generalization performance for low-sample and low signal-to-noise ratio environments.

Within supervised learning pipelines, eXtreme Gradient Boosting (XGBoost) and LSTM stand for two divergent technical paradigms: tree-based ensemble algorithms featuring strong interpretability, fast training speed and decent robustness against outliers; and recurrent neural network-based deep learning architectures that support automated feature extraction and long-range temporal dependency capture while suffering from poor interpretability.

Özbey et al. [58] benchmarked the two algorithms against 13+ year GNSS records from 15 stations across Anatolia. Their results show that XGBoost attains a trend-fitting R^2^ of 0.84, marginally outperforming LSTM (0.81). When identifying co-seismic offsets under 10% outlier contamination, both techniques deliver detection accuracy above 80%.

This observation carries meaningful implications: ensemble learning can rival deep learning for GNSS long-term trend decomposition, and model complexity does not monotonically correlate with prediction precision. The underlying mechanism is that GNSS long-term trends are dominated by low-frequency constituents. XGBoost’s hierarchical splitting logic inherently fits large-scale slow variations, while the full sequential modeling capacity of LSTM becomes redundant for such low-frequency fitting tasks.

Gabsatarov & Vladimirova [59] verified the cross-setting transferability of machine learning models over three distinct tectonic environments: the Japanese Archipelago, Southern California, and the Peru-Chile margin. Such multi-region validation frameworks are vital to promote practical deployment of machine learning within GNSS geodetic research.

For time series forecasting tasks, Chen et al. [60] put forward the Double VMD-LSTM (DVMD-LSTM) hybrid model. It conducts secondary modal decomposition on residual signals separated by primary VMD to extract oscillatory constituents hidden in residuals. Tested on multi-site GNSS datasets, the algorithm reduces residual RMS by an average of 9.86% and elevates geodetic velocity precision by 33.02%. This outcome verifies that the two-stage decomposition strategy effectively retrieves subtle deformation patterns left unseparated after initial modal extraction.

Synthesizing the findings reported in Özbey et al. [58] and Gabsatarov & Vladimirova [59], we can draw the following preliminary inferences. For core processing tasks including long-term trend fitting and co-seismic offset detection, supervised learning algorithms achieve practically viable performance under ample sample conditions. Their detection precision outperforms basic LS fitting but does not clearly outperform well-tuned MLE.

Accordingly, the intrinsic value of supervised learning within GNSS time series analysis does not consist in replacing conventional processing tools, but manifests in two unique application dimensions. First, it handles complicated geodetic scenarios intractable for classic models, such as post-seismic time series superimposed with multiple successive slow slip or seismic events. Second, it supports automated batch processing across extensive station networks, a workload impossible to accomplish via manual station-by-station interpretation.

The successful detection of deformation signals linked to the Van and Elazig earthquakes documented by Özbey et al. [58] serves as a typical illustration of the first application direction.

Proposed by Teutschmann et al. [18], GNSS-FM (currently an unreviewed arXiv preprint) marks the first formal attempt to deploy the self-supervised time series foundation model paradigm for GNSS coordinate time series processing.

Its technical architecture draws core design logic from wav2vec 2.0 and consists of two key modules. First, it randomly masks roughly 50% of input time steps and trains a Transformer encoder to predict displacement values at masked positions based on contextual information. Second, vector quantization (VQ) maps continuous displacement constituents onto a discrete codebook containing 128 codewords, enabling the model to learn compact discrete representations of geodetic time series.

Pre-trained on datasets collected from more than 17,000 global stations, this foundation model boosts displacement prediction accuracy by 88.5% relative to supervised learning baselines; its earthquake detection performance also outperforms supervised benchmarks significantly. Note that this performance metric originates from an unreviewed preprint and awaits formal peer review for validation.

The fundamental distinction separating GNSS-FM from classic time series decomposition tools lies in their operating logic. SSA and VMD conduct modal decomposition under mathematical premises of signal orthogonality and narrowband limitation, while GNSS-FM learns the implicit mapping between deformation constituents and stochastic noise purely from observational data. In theory, this data-driven paradigm circumvents two inherent drawbacks of conventional decomposition algorithms: mode mixing and hyperparameter sensitivity.

Even so, GNSS-FM faces four prominent practical bottlenecks.

a. Cross-regional generalization bias.

Its pre-training corpus is dominated by sites across North America, Europe and East Asia. Its performance over data-sparse geodetic regions (Africa, South America, Central Asia) remains unvalidated, and the geographical imbalance of existing global observation networks may be encoded as biased latent representations within the model.

b. Lack of physical interpretability.

No clear one-to-one correspondence exists between discrete codewords in the VQ codebook and established geophysical signals, such as annual periodic constituents and co-seismic jumps, which obstructs geophysical interpretation of model outputs.

c. Limited complementarity with traditional decomposition schemes.

Though fine-tuning for downstream tasks substantially elevates GNSS-FM’s performance, its zero-shot generalization capacity on unlabeled raw time series (e.g., initial measurements from newly deployed stations) remains undetermined compared with training-free SSA for blind unsupervised decomposition.

d. Unverified reproducibility of preprint outcomes.

Since the relevant research has not undergone rigorous peer review, additional tests on independent geodetic datasets and multi-scale GNSS network configurations are required to verify whether the reported 88.5% accuracy improvement can be stably reproduced, a critical criterion for judging its practical applicability.

## 4. Geophysical Interpretation of Time-Varying GNSS Components

Nonlinear long-term trends, seasonal periodic constituents, transient deformations and spatially correlated common-mode errors embedded within GNSS coordinate time series are not mere stochastic observational noise. Instead, they constitute integrated surface deformation responses induced by multi-scale geophysical processes across diverse spatial and temporal ranges.

Based on the signal characteristics and decomposition approaches elaborated on in Section 3, this chapter conducts systematic interpretation of the geophysical driving mechanisms governing all categories of time-varying geodetic constituents and builds an intrinsic linkage between geodetic measurements and crustal geodynamic evolution [5,13].

### 4.1. Nonlinear Long-Term Constituents and Regional Crustal Deformation

Long-term geodetic constituents extracted from GNSS coordinate time series serve as direct proxies for crustal motion across large spatiotemporal scales. Linear trends mainly record coherent plate motion and stable tectonic deformation, whereas nonlinear long-term signatures arise from multiple coupled factors: unsteady tectonic evolution, elastic and viscoelastic crustal responses induced by surface mass redistribution, and anthropogenic disturbances.

Horizontal secular variations are predominantly governed by tectonic activity, while vertical long-term deformation is more susceptible to surface mass loading and human-induced geomorphic changes.

Globally, secular linear horizontal constituents retrieved from GNSS time series arise from large-scale lithospheric plate motion, acting as the fundamental observational constraint for constructing and maintaining the ITRF [3].

Argus et al. [61] combined geological and geodetic measurements to develop the NNR-MORVEL56 plate motion model covering 56 major tectonic plates. Their outcomes validate that GNSS can precisely resolve millimeter-scale interplate displacements.

For mainland China, Zhang et al. [62] analyzed velocity fields derived from CMONOC stations and systematically characterized the bulk crustal motion driven by the India-Eurasia collision.

Within plate boundary zones, accelerated variations in nonlinear long-term signatures generally correlate tightly with pre-seismic strain buildup [63].

Nonlinear secular vertical constituents derived from GNSS coordinate time series are jointly controlled by four geophysical and anthropogenic drivers: tectonic activity, GIA, surface mass redistribution, and human subsurface exploitation.

Tectonic vertical differential deformation takes the form of heterogeneous nonlinear uplift and subsidence across fault boundaries, which is tightly coupled with lower crustal flow dynamics [64,65]. Glacial isostatic adjustment arises from crustal viscoelastic rebound triggered by the retreat of paleo-ice sheets. Within formerly glaciated domains of North America and Northern Europe, GNSS-measured vertical uplift velocities peak at 10 mm/yr. Milne et al. [26] leveraged multi-year GNSS observational records to constrain layered mantle viscosity structures.

In terms of surface mass loading variations, accelerated nonlinear subsidence induced by groundwater over-extraction dominates vertical nonlinear deformation in densely populated human-modified regions [9]. For aquifer overexploitation zones including the North China Plain, a joint processing framework integrating VMD, SSA and GRACE gravity datasets achieves quantitative decomposition of tectonic subsidence and compaction-driven subsidence from groundwater depletion.

### 4.2. Geophysical Excitation Mechanisms of Periodic Geodetic Constituents

Annual and semi-annual geodetic constituents constitute the most striking time-varying signatures embedded within GNSS coordinate time series, featuring evidently variable amplitudes and phases over time.

Throughout this section, each quantitative figure is reported together with the metric it refers to (variance or residual-power reduction, amplitude ratio, RMS or WRMS reduction). These quantities are mathematically related but not interchangeable, and cross-study comparisons should respect the metric differences.

Blewitt & Lavallée [19] first systematically verified that omitting periodic deformation components introduces significant biases into station velocity solutions. Such deviations can reach millimeters per year if the time span of observational records is shorter than 2.5 years.

This periodic deformation originates from a core geophysical framework: elastic Earth loading theory. Periodic redistribution of atmospheric, hydrological and cryospheric mass imposes time-dependent surface loads on the solid Earth, thereby generating elastic crustal deformation responses [57].

Van Dam et al. [13] conducted the first systematic comparisons between European GNSS measurements and vertical crustal displacements inverted from GRACE gravity data. Their findings confirm that environmental mass loading acts as the primary driver of vertical periodic constituents recorded by GNSS.

For the excitation mechanisms of annual and semi-annual geodetic constituents, Dong et al. [6] analyzed seasonal terms at 128 globally distributed sites. Their results show that most vertical annual amplitudes are below 10 mm (typically 4–10 mm), with a weighted mean amplitude of 5.47 mm before pole tide correction, decreasing to 4.19 mm after pole tide correction and to 3.19 mm after mass loading correction.

Hydrological loads including soil moisture, snowpack and groundwater dominate vertical periodic deformation across most continental areas. Van Dam et al. [66] modeled crustal displacements induced by continental water storage and compared them with monthly GPS heights at 147 globally distributed sites; after the modeled hydrological displacements are removed, the variance of the GPS height time series is reduced, on average, by an amount comparable to the variance of the modeled displacements, with variance reductions observed at 92 of the 147 sites; at the 23 sites whose observed annual phase agrees with the model, the modeled annual amplitude amounts to 25–100% of the observed annual amplitude. After polar tide correction, the combined influences of atmospheric, non-tidal oceanic and hydrological loads explain about 42% of the residual power of annual deformation signals and reduce the weighted mean vertical annual amplitude from 4.19 mm to 3.19 mm at 90 of the 128 analyzed sites [6]. Snow coverage and permafrost thaw act as major contributors in high-latitude and high-elevation zones, whereas non-tidal ocean loading serves as a critical excitation source for coastal sites [16]. Full load correction integrating atmospheric, hydrological and non-tidal oceanic terms markedly reduces the RMS of GNSS vertical constituents.

In regional investigations within mainland China, Jiang et al. [15] discovered that four categories of surface mass loads lower the weighted root mean square (WRMS) of vertical annual amplitudes for only 80% of stations. Hu et al. [67] verified that hydrological mass loading constitutes the primary trigger for vertical annual cyclic variations recorded by GNSS in Yunnan Province.

In terms of quasi-periodic variations, intense El Niño episodes over the Amazon Basin trigger interannual vertical deformation anomalies of 20–30 mm in regional GNSS time series [68]. Such time-dependent periodic signatures place strict demands on signal decomposition algorithms. Klos et al. [27] analyzed 26 IGS reference stations and found that over 70% of these sites show interannual standard deviations of annual amplitude fluctuations exceeding 0.5 mm, rendering conventional fixed harmonic fitting incapable of satisfying precision criteria. Nonparametric schemes represented by VMD can retrieve time-varying annual constituents efficiently; Section 3.2 elaborates on their algorithmic principles and comparative performance.

By investigating continuous GPS measurements in northwestern Yunnan, Zhang et al. [69] further verified the significant impact of surface mass loading on CME filtering. Their study compared two CME filtering strategies and demonstrated that removing local topographic and hydrological effects before spatial filtering substantially improves the filtering performance. Combining hydrological loading derived from GRACE with atmospheric pressure and ocean mass loading data, the vertical RMS reduction increased from 15.19% to 33.4%, and the efficiency metric improved from 0.28 to 0.55. This underscores the importance of accounting for site-specific loading contributions, particularly seasonal hydrological mass redistribution in regions with complex terrain, when performing CME mitigation in regional GNSS networks.

There remains a long-standing research puzzle spanning over two decades: environmental mass loads explain only a small fraction of the observed horizontal annual amplitude [15]. Four dominant hypotheses have been put forward to fill this theoretical gap, but none have received quantitative validation to date.

a. Thermoelastic horizontal deformation hypothesis.

Horizontal projections of thermoelastic displacement arising from bedrock and monument foundations may be larger than previously predicted. However, horizontal outputs from existing TED models [8] suffer from low precision due to uncertain thermophysical input parameters.

b. Terrain–atmosphere coupling hypothesis.

Complex topography exerts localized modulation on atmospheric loading responses, and such modulation effects are amplified within horizontal deformation records. Conventional global load models adopt coarse spatial resolutions of 0.5–1°, which cannot fully capture small-scale topographic coupling signals.

c. Processing artifact hypothesis.

Unmodeled systematic biases embedded in GNSS orbit and clock products introduce spurious annual periodic artifacts into horizontal time series, with the east–west component being most severely contaminated.

d. Undersampled near-surface loading hypothesis.

Near-surface hydrological cycles (e.g., seasonal freeze–thaw of soil moisture) and lateral groundwater migration create horizontal load gradients that are absent from current coarse-resolution global load databases.

Each conjecture gains fragmentary observational evidence but lacks comprehensive systematic validation. Resolving this open problem is one of the most critical cutting-edge directions for future GNSS physical modeling research.

Lu et al. [8] released a global temperature-forced TED dataset (2000–2023), which addresses a critical gap in quantitative characterization of thermoelastic geodetic constituents.

Preliminary tests across global IGS stations indicate that TED can explain 20–35% of residual scatter in GNSS time series, suggesting that thermoelastic deformation may constitute a non-negligible geophysical component. We emphasize that the TED dataset is currently an ESSD Discussions preprint [8] that has not yet been peer-reviewed; these percentages should therefore be regarded as preliminary indications pending formal validation.

Even so, TED’s reliability is constrained by two core drawbacks.

First, inherent uncertainties exist within thermophysical parameters. The TED model adopts thermal conductivity, heat capacity and other properties extracted from the SoilGrids global soil database; these parameters are spatially interpolated via machine learning from sparse in situ soil profile measurements.

In data-scarce zones including inland Africa, Central Asian plateaus and deep Amazon rainforest, SoilGrids parameter uncertainties range from 30% to over 50%, and such errors propagate linearly to all TED displacement outputs.

Second, the geometric simplifications embedded in the model introduce systematic bias. TED’s layered finite-element framework presumes horizontally stratified subsurface media and fully anchored monument foundations connected to bedrock. For plains and basins with thick sedimentary cover, sediments feature low thermal conductivity and heterogeneous porosity that distort heat transfer paths, thereby triggering consistent overprediction of thermoelastic displacements by TED.

Lu et al. [8] explicitly advised that TED only serves as a preliminary correction term instead of a definitive geophysical compensation product. In practical post-processing workflows, however, TED is frequently applied directly as a standard correction without prior regional validation.

A feasible optimization scheme is to attach site-wise confidence bounds to TED datasets, generated by Monte Carlo uncertainty propagation relying on local input parameter errors.

### 4.3. Transient Geodetic Constituents and Seismic Cycle Research

Transient geodetic constituents extracted from GNSS coordinate time series evolve over timescales ranging from days to years, with origins stemming from both tectonic and non-tectonic geophysical processes [20]. According to their distinct temporal deformation patterns, such transients fall into three categories: step offsets (co-seismic jumps), decaying signals (post-seismic relaxation), and creeping gradual variations (slow slip events).

Co-seismic geodetic constituents manifest as distinct step-like transient offsets within GNSS coordinate time series, and their spatial deformation fields act as fundamental observational constraints for inverting distributed fault-slip rupture models [70,71].

Taking the 2008 Mw 7.9 Wenchuan earthquake as a representative case, Xu et al. [70] derived a total seismic moment of 8.74 × 10^20^ Nm (equivalent to Mw 7.90) via inversion relying on co-seismic displacement measurements from CMONOC stations. Permanent east–west co-seismic offsets of roughly 60 cm were recorded at geodetic sites located on the fault hanging wall [71].

For the 2011 Mw 9.0 Tohoku-Oki subduction earthquake, peak horizontal co-seismic displacements exceeding 20 m were detected across the epicentral rupture zone, with a maximum eastward offset of 5.3 m recorded along the Pacific coastal margin [72].

Post-seismic geodetic constituents arise from three coupled geophysical mechanisms: afterslip on fault planes, viscoelastic relaxation within the lower crust and upper mantle, and pore-fluid pressure diffusion [29,30].

For post-seismic signals triggered by the Wenchuan earthquake, Cao et al. [29] inverted cumulative deformation fields spanning post-event years 6 to 11 using Sentinel-1 InSAR measurements. Their results verify that near-fault deformation is governed by fault afterslip, whereas viscoelastic relaxation dominates mid- and far-field crustal motion.

Liu et al. [30] built a 3D viscoelastic finite-element model constrained by GNSS post-seismic time series, which quantitatively resolved the viscosity of the mid-to-lower crust beneath the eastern Tibetan Plateau.

Post-seismic transients recorded after the Tohoku-Oki earthquake similarly display combined signatures of fault afterslip and mantle viscoelastic relaxation [72,73].

Slow-slip events (SSEs) represent the dominant transient tectonic geodetic constituents along plate boundary seismic zones. These events mostly take place at subduction plate interfaces or active fault planes, generating surface displacements ranging from millimeters to centimeters without detectable radiated seismic waves [50,74].

Dragert et al. [28] first discovered periodic SSEs within the Cascadia subduction zone, which endure 1–2 weeks and recur roughly every 14 months with equivalent moment magnitudes of Mw 6.5–7.0.

For the Japanese Nankai Trough, recent surveys have captured shallow SSEs lasting ~118 days. Their slip patterns show spatial complementarity with locked patches prone to large megathrust earthquakes [75].

From a geodynamic perspective, Wang et al. [76] newly identified secondary acceleration of slip fronts induced by coalesced SSE clusters in subduction margins. This finding supplies novel mechanical constraints to interpret cascaded SSE triggering and interseismic stress redistribution across fault interfaces.

The methodological evolution spanning parametric curve fitting [28], multi-station joint spectral decomposition [48], and automated transient detection via machine learning [49] embodies a full paradigm shift: from validating known slow slip events to discovering unrecognized transient geodetic constituents.

Nevertheless, this transition brings significant inconsistencies in cross-method comparison. The hyperbolic tangent fitting model proposed by Dragert et al. [28] is built upon rigorous physical understanding of Cascadia subduction SSEs, specifically frictional slip mechanics along the plate boundary interface. By contrast, machine learning detection architectures developed by Tanaka et al. [49] may deliver higher detection accuracy but operate as opaque black boxes without traceable decision pathways.

When ML outputs and physics-based parametric models yield starkly divergent estimates of SSE onset timing or total event duration, reconciling the two sets of results lacks objective evaluation criteria. The core conflict originates from their inherent limitations: data-driven ML lacks interpretable physical logic, while conventional parametric models are tightly constrained by simplified theoretical assumptions.

This persistent validation dilemma between novel data-driven algorithms and established physics-grounded benchmarks is a widespread but understudied bottleneck hindering the integration of machine learning into geophysical interpretation of GNSS time series.

Regarding non-tectonic transient geodetic constituents, a variety of non-tectonic geophysical processes, including extreme hydrological episodes, reservoir impoundment, and volcanic unrest, can generate pronounced transient surface deformation.

Nahmani et al. [10] successfully detected vertical transient geodetic constituents driven by flood inundation along the Niger River in West Africa. Han et al. [77] demonstrated that intense precipitation linked to the 2010–2011 La Niña event produced broad-scale elastic subsidence of ~10 mm across the Australian continent. Subsequent terrestrial water storage depletion drove gradual crustal rebound throughout the ensuing 3–4 years.

In the Three Gorges area, multi-source geodetic analyses confirm that reservoir filling induces obvious vertical crustal deformation [78]. Dense GNSS monitoring networks deployed atop Kilauea Volcano in Hawaii enable deep learning frameworks to deliver near-real-time forecasting of caldera collapse episodes [79].

Additionally, Nistor et al. [80] processed observational records from around 200 European EPN stations and drew two key conclusions. First, M > 6 earthquakes, landslides and volcanic eruptions not only produce transient offsets but also substantially modulate the amplitude of seasonal geodetic constituents. Second, sites located within 30 km of epicenters show systematically amplified seasonal amplitudes. Two competing physical interpretations remain unvalidated: property variations in shallow subsurface media and modified hydrological loading responses, which require further targeted investigation.

Table 3 provides a systematic comparative summary of co-seismic and post-seismic signatures from typical large earthquakes.

### 4.4. Common-Mode Geodetic Constituents: Spatial Characteristics and Regional Filtering

Spatially correlated CME represent pervasive systematic biases embedded within regional GNSS geodetic constituents. Characterized by synchronous spatiotemporal fluctuations across nearly all stations, CME acts as a dominant error source that hinders high-precision regional crustal deformation monitoring and quantitative geodynamic interpretation.

Wdowinski et al. [84] first introduced the CME definition in 1997. Contemporary research further verifies that such coherent artifacts originate from large-scale geophysical mass processes and unmodeled processing biases, carrying distinct physical driving origins [22,31,32].

CMEs are sorted into three tiers by spatial coverage. Global-scale CMEs stem mainly from ITRF realization uncertainties, satellite orbit biases and geocenter motion [85]. For regional signals spanning hundreds to thousands of kilometers, spatial correlation weakens with growing inter-station separation; the leading principal component of CME across the Sichuan-Yunnan region explains over 70% of total spatial variance [86]. Local-scale coherent variations may arise from unmodelled environmental effects shared by closely spaced stations; strictly site-specific effects such as multipath or monument instability are excluded here because they do not exhibit spatial coherence across stations. Root drivers fall into two broad groups. The first category covers large-scale loading-induced geodetic constituents (atmospheric, hydrological and non-tidal ocean loading). The second refers to systematic processing biases including frame realization discrepancies, orbital inaccuracies and imperfect atmospheric delay parameterization [34].

PCA/EOF decomposition is the most prevalent technique for extracting and eliminating CMEs to date [33]; full elaboration on algorithm fundamentals is provided in Section 3.5.

Wu [4] put forward the PACA. This scheme adopts sub-regional clustered filtering to strengthen the coherence of annual geodetic constituents within each cluster, overcoming the critical drawback of conventional full-domain PCA: genuine regional tectonic transients are prone to being falsely classified as common-mode artifacts.

Ample observational evidence has validated the geodynamic benefits of accurate CME mitigation. After CME filtering, Li et al. [87] successfully detected three distinct slow slip events occurring across Japan’s Boso Peninsula from 2011 to 2019. For the Nepal–Mount Everest zone, Xu et al. [88] reported a maximum 25.5% drop in the RMS of horizontal velocity fields following CME removal, while inversion uncertainties of vertical crustal displacement were constrained below 1 mm/yr.

### 4.5. Geodetic Interpretation Based on Multi-Source Data Fusion

Standalone GNSS measurements carry intrinsic drawbacks including non-uniqueness in geophysical inversion and coarse spatial sampling. Accordingly, joint fusion of multi-type geodetic and geophysical datasets has evolved into the prevailing research paradigm for geodynamic interpretation of transient and periodic geodetic constituents [14].

Multi-source geodetic fusion for time-varying geodetic constituents is built on a core geodynamic principle: complementary observational strengths of distinct geodetic techniques when capturing identical geophysical processes [89]. GNSS provides high-precision three-dimensional point displacement time series with high temporal resolution; GRACE/GRACE-FO provides global mass redistribution observations at a spatial resolution of 400 km; InSAR yields spatially continuous crustal deformation fields, whose temporal sampling is limited by satellite revisit intervals [90].

Three mainstream fusion hierarchies have been established to date. Data-level fusion fills spatial coverage gaps inherent to single-technique observations via joint GNSS–GRACE inversion workflows [91]. Feature-level fusion leverages spatiotemporal decomposition tools such as ICA and EOF to quantitatively separate deformation signals driven by different geophysical load sources. Taking Xinjiang as a typical regional case, Li et al. [92] adopted ICA to isolate hydrologically induced vertical geodetic constituents inverted from GRACE gravity data. After non-tidal atmospheric and ocean load correction, correlation coefficients between GRACE-derived displacements and GNSS vertical transients range from 0.63 to 0.91, accompanied by a weighted RMS reduction of 16.18–58.97%. Model-level fusion constructs a multi-physics coupled inversion framework integrating elastic loading and poroelastic deformation mechanics, which incorporates GNSS, InSAR and GRACE datasets as joint observational constraints to quantitatively resolve terrestrial water storage variations and their associated crustal deformation-driving mechanisms [56].

Recent advances further demonstrate the value of integrating point-wise GNSS measurements with ground-based imaging radar: a three-step data acquisition optimization algorithm for constructing hourly three-dimensional deformation fields by combining GNSS with ground-based InSAR (GNSS-InBSAR) enables high-temporal-resolution deformation-field reconstruction [93], complementing the satellite-based GNSS–InSAR fusion workflows discussed above.

Methodologically, the three-tier fusion workflow outlined above follows a stepwise logical chain from raw observations to interpretable geophysical information.

Data-level fusion relies primarily on joint adjustment and least-squares collocation. Cross-technique observation equations are established to achieve unified simultaneous processing of multi-source geodetic datasets, with dominant error contributions stemming from frame offsets between distinct geodetic techniques and ambiguous weighting schemes for heterogeneous observations.

Feature-level fusion adopts spatiotemporal decomposition algorithms including ICA, EOF and CCA as mathematical operators. Decomposition is executed within feature space instead of raw coordinate space to isolate signals from diverse geophysical drivers. The outstanding bottleneck here concerns the physical interpretability of extracted modes: it remains challenging to map statistically independent components to concrete subsurface or surface geodynamic processes.

Model-level fusion is built around Bayesian joint inversion and multi-physics coupled forward modeling. Physical theories such as elastic loading formulations and poroelastic constitutive equations are embedded as prior constraints, allowing cross-validation of multi-source measurements within a consistent geodynamic framework. Its major limitations consist of low computational efficiency and inherent non-uniqueness across multi-dimensional parameter spaces.

The three fusion tiers are not successive replacement schemes but complementary tools tailored to distinct research objectives. Data-level fusion fits studies with high-quality raw observations and explicit analytical formulations, such as joint reconstruction of co-seismic displacement fields combining GNSS and InSAR. Feature-level fusion is optimal for cases with entangled mixed loading signals requiring blind source separation, for instance, quantifying environmental load contributions to regional CME artifacts. Model-level fusion performs best when governing physical mechanisms are well constrained; however, precise parameter inversion is demanded, e.g., quantifying coupled responses between terrestrial water storage fluctuations and crustal deformation.

At the intersection of machine learning and multi-source geodetic integration, Sorkhabi [94] illustrated promising performance of hybrid ML-geodetic workflows for regional land subsidence evaluation. This work marks an early attempt to transition from purely physics-driven inversion toward a hybrid data-physics co-driven fusion paradigm. Systematization of this three-tier fusion architecture lays the core methodological groundwork to fulfill the long-term goal of a universal unified geodetic inversion framework [14].

In parallel, when GNSS observations are fused with complementary sensors, inaccurate temporal synchronization can introduce artificial transients and reduce the reliability of time-varying signal extraction, so rigorous time alignment is a prerequisite for trustworthy multi-source fusion [95].

In global hydrological cycle research, Bevis et al. [96] deployed GNSS monitoring stations across the Amazon Basin and observed annual peak-to-peak vertical geodetic constituents of 50–75 mm at the Manaus (NAUS) site. Such vertical transients show strong negative correlation with river water levels with zero time lag, providing the first definitive proof that GNSS can capture elastic crustal deformation driven by fluvial mass redistribution, and laying a fundamental observational foundation for hydrogeodetic research.

Within seismic geodynamic studies, joint inversion integrating GNSS and InSAR measurements by Shen et al. [82] identified concentrated peak slip at fault intersections for the 2008 Wenchuan earthquake. Yang Yinghui et al. [81] proposed an adjacent-track smoothing constrained correction scheme for InSAR deformation fields; this correction boosted data precision by ~60%, and joint inversion yielded a maximum slip magnitude of ~9.0 m across the Beichuan sedimentary basin.

For the 2011 Mw 9.0 Tohoku-Oki subduction earthquake, Bletery et al. [83] jointly incorporated static GNSS, high-rate GNSS, strong-motion seismograms, teleseismic waveforms and tsunami records to constrain a finite-fault source model featuring a peak slip of 64 m. Their results demonstrate that extreme shallow slip patches cluster tightly along the trench axis. Table 4 systematically summarizes representative multi-source fusion cases combining GNSS, InSAR and GRACE alongside quantitative comparisons of their respective observational performance.

## 5. Discussion

Despite three decades of technical advancement, significant scholarly discrepancies persist across several pivotal unresolved topics in GNSS time series analysis. Specifically, standardized parameter-selection guidelines remain absent for nonparametric decomposition algorithms [24,97,98]; the physical origins of common-mode errors (CMEs) remain debated [22,31]; environmental loading models explain only a small fraction of the horizontal annual amplitude [15]; and the black-box nature of deep learning limits physical interpretability [56]. In this section, we re-examine these four controversies against recent publications and identify critical shifts in academic understanding.

It should be noted that several quantitative benchmarks cited in this review, particularly those derived from CMONOC station analyses, originate from specific regional networks. While these results provide valuable large-sample evidence, their generalizability to other geodetic networks (e.g., IGS global stations, GEONET in Japan, SOH in South America) requires further validation. We have made every effort to include complementary results from independent research groups and diverse tectonic settings wherever available.

Re-examining the four core controversies outlined above against recent publications released over the past three years identifies several critical shifts in academic understanding:

First, new insights update debates over parameter tuning criteria for nonparametric decomposition algorithms. Li et al. [17] identified period offsets embedded within GNSS seasonal geodetic constituents, which implies that historical performance benchmarks comparing VMD and SSA under fixed periodicity assumptions [12,24] require systematic re-evaluation. VMD operates with fixed central frequency bands, which inherently causes systematic omission of signals featuring drifting oscillation periods. Its reported RMS reduction across 97.9% of CMONOC stations may partially stem from absorbing genuine frequency-drifted seasonal transients into residual noise. While residual RMS values are lowered, the complete extraction of time-varying periodic geodetic constituents becomes compromised. Numerical results from the UKF_Amp_Period model provide indirect supporting evidence for this inference: this method improves fitting precision by 61% relative to weighted least squares (WLS) and by 40.6% compared with standard SSA [17]. However, independent controlled validation experiments are still required to fully corroborate this deduction. Accordingly, the 97.9%/69.8% benchmark cited in this review must be interpreted with caution: it is single-network (CMONOC), self-sourced from the authors’ prior work, and awaits independent replication; residual RMS improvement also does not by itself guarantee complete recovery of time-varying periodic constituents.

Second, fresh progress reshapes debates surrounding the physical origins of spatially correlated CMEs. The public release of the TED dataset (currently an ESSD Discussions preprint [8]) provides a novel quantitative tool to isolate thermoelastic signals embedded within common-mode artifacts. If regional CME signatures partially arise from coherent large-scale thermoelastic crustal deformation, post-TED-residual CME components will dominantly retain only systematic processing biases. This creates a physics-based discrimination workflow to untangle the dual-source ambiguity of CMEs. That said, intrinsic uncertainties inherent to TED products (see Section 4.2) restrict this strategy to delivering merely qualitative clues rather than robust quantitative judgments.

Third, updated insights reshape ongoing debates over the applicability of machine learning workflows. The advent of GNSS-FM [18] elevates the core academic dispute to a new dimension: the original question of whether ML is valid for GNSS geodetic constituent analysis has largely been answered in the affirmative, while a critical open question emerges: can self-supervised pre-training paradigms restructure the entire disciplinary method ecosystem? A growing body of studies [49,58,59] has demonstrated the practical viability of data-driven ML for GNSS time series processing, providing substantial—though not yet unconditional—support for the former question; the open weaknesses listed above (interpretability, sample dependence, generalization) indicate that this support is best described as encouraging rather than definitive. In contrast, the latter question remains unresolved, with its final judgment hinging on the degree of progress across four core research challenges: cross-regional generalization capacity, traceable physical interpretability, geographic fairness of pre-training datasets, and reproducibility of preliminary preprint outcomes (see Section 3.6 for comprehensive elaboration). From a short-term perspective (3–5 years), a pragmatic outlook anticipates self-supervised foundation models acting as an auxiliary supplementary module embedded within existing mature algorithm frameworks. Such models can yield marginal performance improvements for tasks with abundant labeled geodetic observations, but they cannot fully replace decades-long validated physics-constrained conventional approaches.

Fourth, new critical reflections have emerged regarding the interdisciplinary integration between geodesy and hydrology. Li et al. [5] and White et al. [99] independently delivered comprehensive review papers summarizing the geodetic signatures induced by environmental loading from geodetic and hydrological disciplinary perspectives, respectively. Cross-comparison of these two syntheses reveals a subtle divergence in core research logics. Li et al. [5] centered their discussion on the corrective performance of loading models for raw GNSS geodetic constituents, following a geodesy-driven paradigm where physical load formulations refine observational biases. By contrast, White et al. [99] framed the problem from a hydrological standpoint, focusing on how GNSS-measured crustal displacements serve as hard constraints to calibrate terrestrial hydrological model parameters, representing an observation-for-model hydrological workflow.

This fundamental contrast originates from distinct core disciplinary objectives. Geodesists adopt environmental loading models to suppress residual noise within GNSS time series; hydrologists utilize GNSS vertical and horizontal deformation signals to invert spatiotemporal variations in terrestrial water storage. Substantial methodological gaps separate the two communities in two critical aspects: the propagation scheme of observational uncertainty and standardized frameworks for model validation. For instance, geodesy routinely quantifies correction efficacy via percentage reduction in RMSE, while hydrological research prioritizes the credible uncertainty bounds of inverted subsurface hydrological parameters. Such inherent disciplinary discrepancies cannot be eliminated merely through open data sharing; collaborative cross-field research is required to reach unified consensus on universal validation benchmarks.

## 6. Conclusions and Future Outlook

### 6.1. Key Conclusions

This paper presents a systematic comprehensive synthesis of existing studies targeting extraction algorithms and geodynamic interpretation for time-varying geodetic constituents embedded within GNSS coordinate time series. A complete hierarchical research architecture is established across four core dimensions: modeling frameworks, spatiotemporal signal signatures, signal decomposition workflows, and underlying geophysical driving mechanisms. Based on a broad collation and comparative analysis of three decades of relevant geodetic publications, the core conclusions of this review are summarized as follows:

(1) The modeling framework has undergone continuous refinement. The general functional model for GNSS coordinate time series has evolved from an elementary formulation that only incorporates linear trends and stationary periodic oscillations to a full-spectrum component model covering linear kinematic trends, seasonal periodic signals, abrupt position offsets, and nonlinear post-seismic crustal deformation. This framework explicitly characterizes four categories of deterministic geodetic constituents and two types of stochastic noise, laying a solid theoretical foundation for signal decomposition and geodynamic interpretation of GNSS observational data [1,2,3].

(2) The multi-layered methodological system has been well established, yet fundamental bottlenecks persist across all categories. MLE is the recognized standard routine for steady linear geodetic constituent fitting and stochastic noise parameterization, benefiting from its capacity to simultaneously solve deformation kinematic parameters and characterize noise properties [36,37]. In CMONOC benchmark tests reported by Wu et al. [12], VMD yields positive residual RMS improvement at 97.9% of stations; these results originate from our prior work, await independent replication on other networks, and should not be read as guaranteed signal recovery (Section 5). Pre-trained on over 17,000 global GNSS sites, the self-supervised GNSS-FM (currently an unreviewed preprint) reportedly boosts displacement prediction precision by 88.5% relative to supervised baseline algorithms, demonstrating promising—yet to be independently reproduced—prospects for self-supervised learning within GNSS time series analysis [18].

Nevertheless, standardized benchmarks for fair cross-algorithm evaluation remain absent. No single technique can simultaneously satisfy three core performance demands: high measurement precision, robust stability, and traceable physical interpretability. Furthermore, the period-drift phenomenon reported by Li et al. [17] at ten CMONOC stations points to a potential shared limitation of decomposition schemes built on fixed periodicity assumptions: if confirmed on wider networks, such drift may introduce unquantified systematic biases by disregarding drifting oscillatory signals embedded within raw geodetic observations.

(3) The geophysical driving mechanisms of GNSS geodetic constituents have been progressively constrained, but critical unresolved knowledge gaps persist. Long-term horizontal kinematic transients originate predominantly from plate motion and tectonic strain accumulation [61]. Vertical periodic oscillations are governed by three major surface load sources: atmospheric mass loading, terrestrial hydrological storage variation, and non-tidal oceanic loading. Their combined contribution explains about 42% of the residual power of the observed annual vertical signal after pole tide correction, reducing the weighted mean vertical annual amplitude from 4.19 mm to 3.19 mm [6], and TED is suggested by preliminary (not yet peer-reviewed) tests to be a potentially important secondary load component [8].

However, environmental load models explain only a small fraction of the observed horizontal annual oscillation amplitudes [15]. This long-standing research blank, recognized since the early global analyses of GPS seasonal signals [6], still lacks quantitative verification from any of the four prevailing explanatory hypotheses: horizontal thermoelastic deformation signals, topographic coupling responses, common-mode artifacts induced by data processing, and uncharacterized minor load sources.

(4) Multi-source geodetic fusion has delivered promising preliminary progress. Integrated measurements combining GNSS, InSAR and GRACE effectively offset inherent limitations of individual geodetic techniques in three aspects: spatial resolution, temporal sampling and observational dimensionality. Joint GNSS–GRACE inversion addresses the problem of insufficient spatial coverage of ground monitoring networks [91]. After non-tidal atmospheric and ocean load correction, the correlation coefficient between GRACE-derived vertical geodetic constituents and GNSS vertical displacements ranges from 0.63 to 0.91 [92]. For seismic case studies covering the 2008 Wenchuan earthquake and the 2011 Tohoku earthquake, joint GNSS–InSAR inversion enables a paradigm shift from simple single-field deformation depiction to quantitative geodynamic interpretation of multi-physics coupled tectonic mechanisms [14,81,82,83].

(5) Fundamental disciplinary challenges continue to persist. Contemporary geodetic investigations are confronted with four prominent bottlenecks. First, full decoupling of mixed multi-source geodetic constituents cannot be achieved; aggregated environmental loading models explain only about 42% of the residual power of the vertical annual signal (reducing the weighted mean amplitude from 4.19 mm to 3.19 mm [6]), and an even smaller fraction of the horizontal annual amplitude [15]. Second, all existing algorithmic workflows have inherent structural defects [27,41,97]. Third, major discrepancies exist between vertical displacement datasets released by different institutions [16]. Fourth, global geodetic research suffers from uneven spatial distribution of monitoring and case studies [64,100].

### 6.2. Future Outlook

To address the pivotal bottlenecks restricting existing geodetic research and align with cutting-edge technological trends across geodesy and geodynamics, five priority research avenues are outlined for future exploration.

(1) Develop physics-informed intelligent algorithms for time-varying geodetic constituent extraction. Geophysical priors, including environmental loading deformation theories and elastic mechanical governing equations, can be embedded as soft regularization terms within deep learning loss functions. After resolving the weighting trade-off between physical constraint penalties and data fitting residuals, physics-constrained neural network architectures such as PINNs can be established. This framework improves traceable physical interpretability for model outputs and enables seamless integration of data-driven learning with geophysical theoretical constraints [56].

(2) Formulate a complete generalized theoretical paradigm for joint inversion and fusion of multi-source geodetic observations. Bayesian statistical inference frameworks should be advanced to facilitate integrated inversion of heterogeneous geodetic datasets, systematically resolving three core technical obstacles: weight assignment for disparate measurement sources, full uncertainty propagation, and rigorous quantitative uncertainty estimation [14].

(3) Advance high-fidelity regional environmental loading models. Current global load datasets suffer from prominent accuracy degradation when applied to local-scale research. To mitigate this limitation, high-resolution hydrological, meteorological and topographic geospatial datasets should be incorporated to tackle core bottlenecks in fine-scale quantitative modeling: synchronous hydrological loading responses and thermoelastic thermal expansion deformation cycles [78,101].

(4) Reinforce the construction of geodetic observation networks and multi-scale collaborative research in data-sparse regions. Active geodynamic domains with severely limited observational coverage, including the Tibetan Plateau, Central Africa and the Amazon Basin of South America, require densified continuous GNSS station networks. Combined with wide-area spatially continuous deformation mapping from InSAR and GRACE satellite gravity constraints on global mass redistribution, coordinated multi-scale crustal deformation analysis spanning local to global extents can be performed [64,100]. Meanwhile, unified global processing standards and standardized product formats for GNSS time series should be promoted to build an open-access shared global geodetic time series repository [1].

(5) Advance real-time GNSS geodetic constituent extraction techniques tailored for geological hazard early warning applications. Novel algorithms targeting high-frequency real-time signal decomposition and autonomous anomaly discrimination should be developed. A multi-technique integrated real-time monitoring and early warning framework can be constructed by combining GNSS receivers, seismometers and InSAR measurements, which strengthens the rapid emergency response and predictive warning performance against abrupt geological disasters including seismic events, volcanic unrest and slope landslides [46,79].

## Figures and Tables

**Table 1:** Comprehensive comparison of major signal extraction methods.

Method Category	Representative Algorithms	Applicable Signal Types	Core Advantages	Major Limitations	Key Performance Metrics	Evaluation Dataset
Parametric	LS, MLE	Stationary linear trends, periodic signals with fixed frequencies	Clear physical interpretation, high computational efficiency	Poor adaptability to non-stationary signals; bias introduced by conventional fixed-period assumptions (interannual annual-cycle fluctuation: ±2 days) [17]	Up to 5–10× underestimation of velocity uncertainty when colored noise is present but a white-noise covariance is assumed; corrected by MLE with an appropriate noise model [11]	Global GPS stations [11]
Nonparametric time-domain decomposition	SSA, EMD/EEMD/CEEMDAN, VMD	Time-varying periodic signals, nonlinear trends	Adaptive operation, no predefined model requirement	Subjective parameter tuning, weak physical interpretability	Positive RMS improvement at 90.5% of stations via SSA [24]; positive improvement at 97.9% of stations via VMD [12] (CMONOC benchmark, same research group; independent replication pending)	CMONOC regional network, China [12,24]
Semiparametric	KF/AKF, spline fitting, compensated least squares	Unidentified transient components, nonlinear trends	Balances explicit physical meaning and adaptive capacity	Performance hinges on prior assumptions embedded in the state-space model	—	—
Spatiotemporal filtering	PCA/EOF, ICA, MSSA	Spatially correlated common-mode errors (CME)	Multi-station joint processing, effective CME suppression	Risk of misinterpreting regional crustal deformation signals	PCA RMS reduction: 51.9–59.1%; ICA RMS reduction: 26.9–44.7% (Antarctic Peninsula network) [35]	Antarctic Peninsula GNSS network [35]
Machine learning	GPR, LSTM/GRU, Transformer, PINN	Complex nonlinear signals analyzed with large datasets	Automated feature learning, strong prediction accuracy	Low model interpretability, requirement for massive training samples	—	—

The performance metrics listed in this table were obtained on different networks with different station counts and evaluation protocols (see the “Evaluation Dataset” column); they are therefore indicative rather than directly comparable and should not be read as a universal ranking of the methods.

**Table 2:** Decision matrix for method selection.

Signal Type	Accuracy Priority	Computational Efficiency Priority	Interpretability Priority
Stationary linear trend	MLE	LS rapid fitting	MLE coupled with noise models
Time-varying periodic signal	VMD > CEEMDAN (CMONOC benchmark [12])	SSA (large window size)	VMD (frequency-domain constraints)
Transient/step offset (known timing)	Parametric Heaviside function + logarithmic/exponent terms	Heaviside step function	Parametric fitting
Transient event (unknown timing)	AKF	SSA offset detection	Kalman filtering
Long-term nonlinear trend	Low-frequency components extracted via VMD	Spline fitting	Compensated least squares
Spatially distributed CME	ICA/MSSA	PCA/EOF	ICA (independent source interpretation)
Large-sample multivariate coupled signals	Transformer > PINN	GPR	PINN (physics-informed constraints)

The accuracy-priority rankings in this table reflect benchmark results mainly from the CMONOC network [12,24]; cross-network generality of the implied ordering has not been validated.

**Table 3:** Comparison of GNSS co-seismic and post-seismic deformation features across representative seismic events.

Event	Occurrence Time	Mw	Seismogenic Zone	Peak Surface Co-Seismic Displacement	Peak Slip Magnitude	Key Geophysical Parameters	Key References
Wenchuan Earthquake	12 May 2008	7.9	Longmen Shan Fault Belt	East–West: 56.08 cm; North–South: 23.9 cm (Maoxian station, strong-motion seismometer)	~9.0 m	Mid lower crust viscosity: 4.0 × 10^18^ Pa·s; Songpan Garzê block lower crust viscosity: 1.5 × 10^18^ Pa·s; seismic moment: 8.74 × 10^20^ Nm	[29,30,70,71,81,82]
2011 Tohoku Earthquake	11 March 2011	9.0	Japan Trench Subduction Zone	Maximum horizontal coastal offset: ~5.3 m; peak horizontal seafloor slip near trench axis: ~50 m (seismic reflection profiling)	50–80 m	Extreme shallow slip concentrated along the trench; peak inverted slip: 64 m (joint inversion)	[72,73,83]
Cascadia Slow Slip Event (SSE)	First detected in 2001	6.5–7.0	Cascadia Subduction Zone	Millimeter to centimeter range	_	Recurrence interval: ~14 months; single episode duration: 1–2 weeks; slip distribution is spatially complementary to megathrust locked zones	[28]

**Table 4:** Typical multi-source fusion cases integrating GNSS, InSAR and GRACE.

Research Case	Fused Data Sources	Fusion Tier	Key Performance Metrics	Core Findings	Key References
Hydrogeodetic Monitoring in Xinjiang	GNSS + GRACE	Data-level/Feature-level	Correlation coefficient between GRACE-inverted deformation and GNSS vertical displacement: 0.63–0.91; weighted RMS reduction: 16.18–58.97%	Consistency between GRACE and GNSS observations improves markedly after non-tidal atmospheric and ocean load correction	[92]
Water Cycle Analysis of the Amazon Basin	GNSS + GRACE	Data-level	Peak-to-peak vertical displacement at the Manaus site: 50–75 mm; strong negative correlation against river stage with zero time lag	GNSS measurements are proven for the first time to resolve elastic crustal deformation induced by fluvial mass redistribution	[96]
Source Inversion of the Wenchuan Earthquake	GNSS + InSAR	Data-level/Model-level	Peak slip magnitude: ~9.0 m; InSAR deformation field correction raises measurement precision by roughly 60%	Peak slip concentrates at fault junction zones within the Beichuan area	[81,82]
Source Characterization of the 2011 Tohoku Earthquake	GNSS, strong-motion seismometer, teleseismic waveform, tsunami records	Data-level/Model-level	Maximum fault slip: 64 m	Extreme shallow slip patches cluster along the trench axis	[83]
Land Subsidence across the North China Plain	GNSS, hydrological gauge, InSAR, groundwater records	Model-level	_	Groundwater over-extraction dominates subsidence driving forces; VMD/SSA paired with GRACE quantitatively separates tectonic subsidence from compaction triggered by groundwater abstraction	[9,91]
Physical Origins of Common-Mode Error (CME) in Taiwan	GNSS (ICA decomposition), ATML, LWS	Feature-level	Correlation of the leading ICA principal component: 0.58 against ATML, 0.40 against LWS	Regional CME signals are predominantly forced by atmospheric mass loading and hydrological loading	[32]

## Data Availability

The original contributions presented in this study are included in the article. Further inquiries can be directed to the corresponding author.

## References

[1] Jiang W.P., Wang K.H., Li Z., Zhou X.H., Ma Y.F., Ma J. (2018). Theory, Methods and Prospect of GNSS Coordinate Time Series Analysis. Geomat. Inf. Sci. Wuhan Univ..

[2] Bevis M., Brown A. (2014). Trajectory Models and Reference Frames for Crustal Motion Geodesy. J. Geod..

[3] Altamimi Z., Rebischung P., Métivier L., Collilieux X. (2016). ITRF2014: A New Release of the International Terrestrial Reference Frame Modeling Nonlinear Station Motions. JGR Solid Earth.

[4] Wu S.G. (2023). Study on Time Series Analysis of CMONOC Stations and Application of Annual Phase-Constrained Clustering Algorithm. Acta Geod. Cartogr. Sin..

[5] Li Z., Jiang W., van Dam T., Zou X., Chen Q., Chen H. (2025). A Review on Modeling Environmental Loading Effects and Their Contributions to Nonlinear Variations of Global Navigation Satellite System Coordinate Time Series. Engineering.

[6] Dong D., Fang P., Bock Y., Cheng M.K., Miyazaki S. (2002). Anatomy of Apparent Seasonal Variations from GPS-derived Site Position Time Series. J. Geophys. Res..

[7] van Dam T.M., Blewitt G., Heflin M.B. (1994). Atmospheric Pressure Loading Effects on Global Positioning System Coordinate Determinations. J. Geophys. Res..

[8] Lu R., Li Z., Ye L., Yuan P., Feng Y. (2026). TED: A Global Temperature-Driven Thermoelastic Displacement Dataset for GNSS Reference Stations (2000–2023). ESSD Discuss..

[9] Li Q., Pu Y.W., Wang Y., Liu Y.C. (2025). Present-Day Crustal Vertical Deformation Characteristics of North China from Dense GNSS Observations. Acta Seismol. Sin..

[10] Nahmani S., Bock O., Bouin M., Santamaría-Gómez A., Boy J., Collilieux X., Métivier L., Panet I., Genthon P., De Linage C. (2012). Hydrological Deformation Induced by the West African Monsoon: Comparison of GPS, GRACE and Loading Models. J. Geophys. Res..

[11] Williams S.D.P. (2003). The Effect of Coloured Noise on the Uncertainties of Rates Estimated from Geodetic Time Series. J. Geod..

[12] Wu S.G., Bian S.F., Li H.P., Li Z., Ouyang H. (2024). Extraction of Time-Varying Signals from GNSS Height Time Series by Variational Mode Decomposition. Acta Geod. Cartogr. Sin..

[13] Van Dam T., Wahr J., Lavallée D. (2007). A Comparison of Annual Vertical Crustal Displacements from GPS and Gravity Recovery and Climate Experiment (GRACE) over Europe. J. Geophys. Res..

[14] Xu G.Y., Wen Y.M., Wang L.Y., Xu C.J. (2025). Current Status and Prospects of Joint Inversion of Geodesy and Geophysics. Geomat. Inf. Sci. Wuhan Univ..

[15] Jiang W.P., Li Z., Liu H.F., Zhao Q. (2013). Cause Analysis of Non-Linear Variation of Coordinate Time Series of IGS Reference Stations in China. Chin. J. Geophys..

[16] Voigt C., Sulzbach R., Dobslaw H., Weise A., Timmen L., Deng Z., Reich M., Stolarczuk N., Peters H., Fietz M. (2024). Non-Tidal Ocean Loading Signals of the North and Baltic Sea from Terrestrial Gravimetry, GNSS, and High-Resolution Modeling. Geophys. Res. Lett..

[17] Li Z., Yang K., Jiang W., Deng L., Zou Y. (2025). Impacts of Period Offset and Period Variation on Modeling Seasonal Signals in GNSS Coordinate Time Series from Both Theoretical and Practical Perspectives. Geo-Spat. Inf. Sci..

[18] Teutschmann N., Crocetti L., Lehmann F., Trentini L., Soja B. (2026). GNSS-FM: A Self-Supervised Foundation Model for Daily GNSS Displacement Time Series. arXiv.

[19] Blewitt G., Lavallée D. (2002). Effect of Annual Signals on Geodetic Velocity. J. Geophys. Res..

[20] Gazeaux J., Williams S., King M., Bos M., Dach R., Deo M., Moore A.W., Ostini L., Petrie E., Roggero M. (2013). Detecting Offsets in GPS Time Series: First Results from the Detection of Offsets in GPS Experiment. JGR Solid Earth.

[21] Song S., Li Y., Hao M., Wang Q. (2026). Extraction and Identification of Transient Deformation after the Ludian Earthquake. Geod. Geodyn..

[22] Li Z., Yue J., Li W., Lu D., Hu J. (2019). Comprehensive Analysis of the Effects of Common Mode Error on the Position Time Series of a Regional GPS Network. Pure Appl. Geophys..

[23] Zhou M.S., Guo J.Y., Shen Y., Kong Q.L., Yuan J.J. (2018). Extraction of Common Mode Errors of GNSS Coordinate Time Series Based on Multi-Channel Singular Spectrum Analysis. Chin. J. Geophys..

[24] Wu S., Li Z., Li H., Bian S., Ouyang H., Yao Y., Peng P., He Y. (2023). Detection of Periodic Signals with Time-Varying Coefficients from CMONOC Stations in China by Singular Spectrum Analysis. IEEE Trans. Geosci. Remote Sens..

[25] Golyandina N., Korobeynikov A., Shlemov A., Usevich K. (2015). Multivariate and 2D Extensions of Singular Spectrum Analysis with the Rssa Package. J. Stat. Soft..

[26] Milne G.A., Davis J.L., Mitrovica J.X., Scherneck H.-G., Johansson J.M., Vermeer M., Koivula H. (2001). Space-Geodetic Constraints on Glacial Isostatic Adjustment in Fennoscandia. Science.

[27] Klos A., Bos M.S., Bogusz J. (2018). Detecting Time-Varying Seasonal Signal in GPS Position Time Series with Different Noise Levels. GPS Solut..

[28] Dragert H., Wang K., James T.S. (2001). A Silent Slip Event on the Deeper Cascadia Subduction Interface. Science.

[29] Cao M.Y., Ji L.Y., Liao X., Liu Y.H., Liu C.J., Shi Y., Zhu L.Y., Xiong G.H. (2025). Post-Seismic InSAR Deformation and Mechanism of the Wenchuan Ms 8.0 Earthquake. J. Geod. Geodyn..

[30] Liu Z.J., Tan K., Wang Q., Wang L., Zhang J., Zhao B., Qiao X.J. (2021). Numerical Simulation of Post-Seismic Afterslip and Viscoelastic Relaxation of the Wenchuan Earthquake. J. Geod. Geodyn..

[31] Li A., Wang Y., Guo M. (2024). Analysis of the Spatial Distribution and Common Mode Error Correlation in a Small-Scale GNSS Network. Sensors.

[32] Feng T., Shen Y., Wang F. (2021). Independent Component Extraction from the Incomplete Coordinate Time Series of Regional GNSS Networks. Sensors.

[33] Dong D., Fang P., Bock Y., Webb F., Prawirodirdjo L., Kedar S., Jamason P. (2006). Spatiotemporal Filtering Using Principal Component Analysis and Karhunen-Loeve Expansion Approaches for Regional GPS Network Analysis. J. Geophys. Res..

[34] Liang B., Yu M.X. (2025). Common Mode Error Analysis of GNSS Coordinate Time Series in Northwest China Based on MSSA Algorithm. Chin. J. Space Sci..

[35] Li F., Li W.H., Zhang S.K., Lei J.T., Zhang Q.C., Yuan L.X. (2019). Spatiotemporal Filtering for Regional GNSS Network in Antarctic Peninsula Using Independent Component Analysis. Chin. J. Geophys..

[36] Williams S.D.P., Bock Y., Fang P., Jamason P., Nikolaidis R.M., Prawirodirdjo L., Miller M., Johnson D.J. (2004). Error Analysis of Continuous GPS Position Time Series. J. Geophys. Res..

[37] Langbein J. (2008). Noise in GPS Displacement Measurements from Southern California and Southern Nevada. J. Geophys. Res..

[38] Cucci D.A., Voirol L., Kermarrec G., Montillet J.-P., Guerrier S. (2023). The Generalized Method of Wavelet Moments with Exogenous Inputs: A Fast Approach for the Analysis of GNSS Position Time Series. J. Geod..

[39] Sun X., Lu T., Hu S., Huang J., He X., Montillet J.-P., Ma X., Huang Z. (2023). The Relationship of Time Span and Missing Data on the Noise Model Estimation of GNSS Time Series. Remote Sens..

[40] Huang N.E., Shen Z., Long S.R., Wu M.C., Shih H.H., Zheng Q., Yen N.-C., Tung C.C., Liu H.H. (1998). The Empirical Mode Decomposition and the Hilbert Spectrum for Nonlinear and Non-Stationary Time Series Analysis. Proc. A.

[41] Qiu X., Wang F., Zhou Y., Zhou S. (2021). Weighted Empirical Mode Decomposition for Processing GNSS Position Time Series with the Consideration of Formal Errors. Acta Geodyn. Geomater..

[42] Dragomiretskiy K., Zosso D. (2014). Variational Mode Decomposition. IEEE Trans. Signal Process..

[43] Li W., Guo J. (2024). Extraction of Periodic Signals in Global Navigation Satellite System (GNSS) Vertical Coordinate Time Series Using the Adaptive Ensemble Empirical Modal Decomposition Method. Nonlin. Process. Geophys..

[44] Ji K., Shen Y., Wang F., Chen Q. (2024). An Efficient Improved Singular Spectrum Analysis for Processing GNSS Position Time Series with Missing Data. Geophys. J. Int..

[45] Fu Y., Fan X., Cao J. (2024). An Imputation Method Based on the Varimax Variant of Multivariate Singular Spectrum Analysis. IEEE Access.

[46] Yang Y., Xu T. (2003). An Adaptive Kalman Filter Based on Sage Windowing Weights and Variance Components. J. Navig..

[47] Vautard R., Ghil M. (1989). Singular Spectrum Analysis in Nonlinear Dynamics, with Applications to Paleoclimatic Time Series. Phys. D Nonlinear Phenom..

[48] Shi K., Chen X., Sun H., Ding H., Guo R., Fan X. (2025). Fine Detection of Transient Signals in GNSS Time Series Using Multi-Station Hankel Spectrum Analysis. JGR Solid Earth.

[49] Tanaka Y., Kano M., Yano K. (2025). Detecting Slow Slip Signals in Southwest Japan Based on Machine Learning Trained by Real GNSS Time Series. JGR Solid Earth.

[50] Rousset B., Bürgmann R., Campillo M. (2019). Slow Slip Events in the Roots of the San Andreas Fault. Sci. Adv..

[51] Yang B., Yang Z., Tian Z., Liang P. (2023). Weakening the Flicker Noise in GPS Vertical Coordinate Time Series Using Hybrid Approaches. Remote Sens..

[52] Chen J., Fang Z., Gao M., Xu Y. (2026). Robust Cooperative GNSS Positioning under Satellite-Constrained Conditions: Accounting for Physical Correlations in Stochastic Modeling. Measurement.

[53] Zhou M., Guo J., Liu X., Shen Y., Zhao C. (2020). Crustal Movement Derived by GNSS Technique Considering Common Mode Error with MSSA. Adv. Space Res..

[54] Li W., Jiang W., Li Z., Chen H., Chen Q., Wang J., Zhu G. (2020). Extracting Common Mode Errors of Regional GNSS Position Time Series in the Presence of Missing Data by Variational Bayesian Principal Component Analysis. Sensors.

[55] Bachelot L., Thomas A., Melgar D., Searcy J., Sun Y.-S. (2025). Cascadia Daily GNSS Time Series Denoising: Graph Neural Network and Stack Filtering. Seismica.

[56] Ma J., Deng Y., Li X., Guo R., Zhou H., Li M. (2025). Recent Advances in Machine Learning-Enhanced Joint Inversion of Seismic and Electromagnetic Data. Surv. Geophys..

[57] Farrell W. (1972). Deformation of Earth by Surface Loads. Rev. Geophys..

[58] Özbey V., Ergintav S., Tarı E. (2024). GNSS Time Series Analysis with Machine Learning Algorithms: A Case Study for Anatolia. Remote Sens..

[59] Gabsatarov Y., Vladimirova I. (2025). Machine Learning for GNSS Time Series Analysis in the Time Domain. Russ. J. Earth Sci..

[60] Chen H., Lu T., Huang J., He X., Yu K., Sun X., Ma X., Huang Z. (2023). An Improved VMD-LSTM Model for Time-Varying GNSS Time Series Prediction with Temporally Correlated Noise. Remote Sens..

[61] Argus D.F., Gordon R.G., DeMets C. (2011). Geologically Current Motion of 56 Plates Relative to the No-Net-Rotation Reference Frame: NNR-MORVEL56. Geochem. Geophys. Geosyst..

[62] Zhang P.Z., Wang M., Gan W.J., Deng Q.D. (2003). Slip Rates of Active Faults Derived from GPS Observations and Their Constraints on Present-Day Continental Geodynamics. Earth Sci. Front..

[63] Huang L., Chen S. (2024). Numerical Simulation of Stress Evolution and Seismic Moment Budget along the Central Segments of the Xianshuihe-Xiaojiang Fault System: Implications for Seismic Hazard Assessment. Tectonophysics.

[64] Liu S., Xu X., Nocquet J.-M., Chen G., Tan X., Jónsson S., Klinger Y. (2025). Lower Crustal Thickening Drives Active Uplift in Northern Tibet. Earth Planet. Sci. Lett..

[65] Rao W.L., Liu B., Tang H., Wang Q.Y., Zhang L., Sun W.K. (2025). Progress in Joint Research of GNSS and GRACE on Crustal Uplift of the Qinghai-Xizang Plateau. Rev. Geophys. Planet. Phys..

[66] Van Dam T., Wahr J., Milly P.C.D., Shmakin A.B., Blewitt G., Lavallée D., Larson K.M. (2001). Crustal Displacements Due to Continental Water Loading. Geophys. Res. Lett..

[67] Hu S.Q., Wang T., Guan Y.H., Yang Z.Y. (2021). Analysis of Vertical Seasonal Fluctuation and Tectonic Deformation in Yunnan Province Based on GPS Measurements and Hydrological Loading Model. Chin. J. Geophys..

[68] Ferreira L., Marotta G.S., Madden E.H., Horbe A.M.C., Santos R.V., Costa J.M.N. (2021). Vertical Displacement Caused by Hydrological Influence in the Amazon Basin. JGR Solid Earth.

[69] Zhang K., Wang Y., Gan W., Liang S. (2020). Impacts of Local Effects and Surface Loads on the Common Mode Error Filtering in Continuous GPS Measurements in the Northwest of Yunnan Province, China. Sensors.

[70] Xu C.J., Liu Y., Wen Y.M. (2009). Inversion of Slip Distribution of the 2008 Wenchuan Mw 7.9 Earthquake Using GPS Data. Acta Geod. Cartogr. Sin..

[71] Liu X.D., Shan X.J., Zhang Y.F., Yin H., Qu C.Y. (2019). Coseismic Deformation Field and Slip Inversion of the Wenchuan Earthquake Based on Strong Motion Records. Seismol. Geol..

[72] Sagiya T., Meneses-Gutierrez A. (2022). Geodetic and Geological Deformation of the Island Arc in Northeast Japan Revealed by the 2011 Tohoku Earthquake. Annu. Rev. Earth Planet. Sci..

[73] Johnson K.M., Fukuda J., Segall P. (2012). Challenging the Rate-state Asperity Model: Afterslip Following the 2011 M9 Tohoku-oki, Japan, Earthquake. Geophys. Res. Lett..

[74] Marill L., Marsan D., Rousset B., Socquet A. (2024). Geodetic Matched Filter Slow Slip Event Detection Along the Northern Japan Subduction Zones. JGR Solid Earth.

[75] Kikuchi Y., Mitsui Y., Kano M. (2025). Detection of Slow Slip Event Lasting Several Months in the Shallow Region of the Suruga Trough, the Eastern End of the Nankai Trough, Japan. Prog. Earth Planet. Sci..

[76] Wang J., Chen K., Michel S., Dal Zilio L., Zhu H., Xia L., Xie J., Hu S. (2025). Secondary Acceleration of Slip Fronts Driven by Slow Slip Event Coalescence in Subduction Zones. Nat. Commun..

[77] Han S. (2017). Elastic Deformation of the Australian Continent Induced by Seasonal Water Cycles and the 2010–2011 La Niña Determined Using GPS and GRACE. Geophys. Res. Lett..

[78] Duan H.R., Zhang C.H., Liang W.K., Wang J.C., Liu P. (2024). Study on Crustal Vertical Deformation Caused by Environmental Loading in the Three Gorges Area Based on Multi-Source Data. J. Geod. Geodyn..

[79] McBrearty I.W., Segall P. (2024). Deep Learning Forecasts Caldera Collapse Events at Kīlauea Volcano. JGR Solid Earth.

[80] Nistor S., Suba N.-S., El-Mowafy A., Apollo M., Malkin Z., Nastase E.I., Kudrys J., Maciuk K. (2021). Implication between Geophysical Events and the Variation of Seasonal Signal Determined in GNSS Position Time Series. Remote Sens..

[81] Yang Y.H., Chen Q., Liu G.X., Cheng H.Q., Liu L.Y., Hu Z.Q. (2014). Smoothing Correction of Adjacent Orbits and Refined Fault Slip Inversion of Coseismic Deformation Field of the Wenchuan Earthquake Using GPS and InSAR. Chin. J. Geophys..

[82] Shen Z.-K., Sun J., Zhang P., Wan Y., Wang M., Bürgmann R., Zeng Y., Gan W., Liao H., Wang Q. (2009). Slip Maxima at Fault Junctions and Rupturing of Barriers during the 2008 Wenchuan Earthquake. Nat. Geosci..

[83] Bletery Q., Sladen A., Delouis B., Vallée M., Nocquet J., Rolland L., Jiang J. (2014). A Detailed Source Model for the Mw 9.0 Tohoku-Oki Earthquake Reconciling Geodesy, Seismology, and Tsunami Records. JGR Solid Earth.

[84] Wdowinski S., Bock Y., Zhang J., Fang P., Genrich J. (1997). Southern California Permanent GPS Geodetic Array: Spatial Filtering of Daily Positions for Estimating Coseismic and Postseismic Displacements Induced by the 1992 Landers Earthquake. J. Geophys. Res..

[85] Wang R., Chen J., Dong D., Tan W., Liao X. (2024). Regional GNSS Common Mode Error Correction to Refine the Global Reference Frame. Remote Sens..

[86] Tan W., Chen J., Dong D., Qu W., Xu X. (2020). Analysis of the Potential Contributors to Common Mode Error in Chuandian Region of China. Remote Sens..

[87] Li M., Yan L., Jiang Z., Xiao G. (2022). Insights into Spatio-Temporal Slow Slip Events Offshore the Boso Peninsula in Central Japan during 2011–2019 Using GPS Data. Geod. Geodyn..

[88] Xu W., Chen G., Ding K., Yang D., Si Y., Yang X. (2022). Analysis of the Common Model Error on Velocity Field under Colored Noise Model by GPS and InSAR: A Case Study in the Nepal and Everest Region. Geod. Geodyn..

[89] Shangguan M., Guo J., Wu S., Zhou X., Zou R., Zhang X. (2025). Joint Inversion of InSAR and GNSS for Surface Subsidence and Terrestrial Water Storage Anomalies of Small-Area in West-Central Yunnan Province, China. J. Hydrol. Reg. Stud..

[90] Carlson G., Werth S., Shirzaei M. (2024). A Novel Hybrid GNSS, GRACE, and InSAR Joint Inversion Approach to Constrain Water Loss during a Record-Setting Drought in California. Remote Sens. Environ..

[91] Li W.Q. (2021). Research on Monitoring of Terrestrial Water Storage Change and Its Loading Deformation by Combined GNSS and GRACE Data. Acta Geod. Cartogr. Sin..

[92] Li W.Q., Guo Q.Y., Zhang C.Y., Wang W., Zhong Y.L., Li W., Xu P.F. (2024). Terrestrial Water Storage and Crustal Vertical Variation in Xinjiang, China Using Independent Component Analysis. Geomat. Inf. Sci. Wuhan Univ..

[93] Liu F., Zhou J., Wang Z., Xu Z. (2026). A Three-Step Data Acquisition Optimization Algorithm for Hourly 3-D Deformation Field Construction Based on GNSS-InBSAR. IEEE Trans. Geosci. Remote Sens..

[94] Sorkhabi O.M. (2025). Fusion of Multi-Source Data and Artificial Intelligence for Land Subsidence Evaluation. Environ. Dev. Sustain..

[95] Li J., Sun R., Wang Y., Ochieng W.Y. (2025). A robust time synchronization algorithm for GNSS/IMU integrated navigation in urban environments. Meas. Sci. Technol..

[96] Bevis M., Alsdorf D., Kendrick E., Fortes L.P., Forsberg B., Smalley R., Becker J. (2005). Seasonal Fluctuations in the Mass of the Amazon River System and Earth’s Elastic Response. Geophys. Res. Lett..

[97] Gao W., Sun Y., Wang L., Wang S. (2022). VMD–WT-Based Method for Extracting On-the-Fly GNSS Tide Level and Its Realization. Remote Sens..

[98] Zhang H., Wang S., Dong C., Xu G., Cai X. (2026). GVMD-NLM: A Hybrid Denoising Method for GNSS Buoy Elevation Time Series Using Optimized VMD and Non-Local Means Filtering. Sensors.

[99] White A.M., Gardner W.P., Borsa A.A., Argus D.F., Martens H.R. (2022). A Review of GNSS/GPS in Hydrogeodesy: Hydrologic Loading Applications and Their Implications for Water Resource Research. Water Resour. Res..

[100] Zhang T., Tian Z., Ji G.F., Li J.Y., Yu X. (2025). Characteristics of Crustal Strain and Vertical Movement in the Northeastern Margin of the Qinghai-Xizang Plateau Based on Continuous GNSS Observations. J. Geod. Geodyn..

[101] Li C., Zhang T., Yang P., He L., Xia Y., Luan W. (2025). Research on the Refinement Algorithm of Surface Loading Deformation Based on Green’s Function. Geod. Geodyn..

